# Raman Study of Pathogenic *Candida auris*: Imaging Metabolic Machineries in Reaction to Antifungal Drugs

**DOI:** 10.3389/fmicb.2022.896359

**Published:** 2022-05-25

**Authors:** Giuseppe Pezzotti, Miyuki Kobara, Tamaki Nakaya, Hayata Imamura, Tenma Asai, Nao Miyamoto, Tetsuya Adachi, Toshiro Yamamoto, Narisato Kanamura, Eriko Ohgitani, Elia Marin, Wenliang Zhu, Ichiro Nishimura, Osam Mazda, Tetsuo Nakata, Koichi Makimura

**Affiliations:** ^1^Ceramic Physics Laboratory, Kyoto Institute of Technology, Kyoto, Japan; ^2^Department of Immunology, Graduate School of Medical Science, Kyoto Prefectural University of Medicine, Kyoto, Japan; ^3^Department of Orthopedic Surgery, Tokyo Medical University, Tokyo, Japan; ^4^Department of Dental Medicine, Graduate School of Medical Science, Kyoto Prefectural University of Medicine, Kyoto, Japan; ^5^The Center for Advanced Medical Engineering and Informatics, Osaka University, Osaka, Japan; ^6^Division of Pathological Science, Department of Clinical Pharmacology, Kyoto Pharmaceutical University, Kyoto, Japan; ^7^Division of Advanced Prosthodontics, The Jane and Jerry Weintraub Center for Reconstructive Biotechnology, UCLA School of Dentistry, Los Angeles, CA, United States; ^8^Medical Mycology, Graduate School of Medicine, Teikyo University, Tokyo, Japan

**Keywords:** *Candida auris*, Raman, antifungal, metabolism, fungal infection

## Abstract

The multidrug-resistant *Candida auris* often defies treatments and presently represents a worldwide public health threat. Currently, the ergosterol-targeting Amphotericin B (AmB) and the DNA/RNA-synthesis inhibitor 5-flucytosine (5-FC) are the two main drugs available for first-line defense against life-threatening *Candida auris* infections. However, important aspects of their mechanisms of action require further clarification, especially regarding metabolic reactions of yeast cells. Here, we applied Raman spectroscopy empowered with specifically tailored machine-learning algorithms to monitor and to image *in situ* the susceptibility of two *Candida auris* clades to different antifungal drugs (LSEM 0643 or JCM15448T, belonging to the East Asian Clade II; and, LSEM 3673 belonging to the South African Clade III). Raman characterizations provided new details on the mechanisms of action against *Candida auris* Clades II and III, while also unfolding differences in their metabolic reactions to different drugs. AmB treatment induced biofilm formation in both clades, but the formed biofilms showed different structures: a dense and continuous biofilm structure in Clade II, and an extra-cellular matrix with a “fluffy” and discontinuous structure in Clade III. Treatment with 5-FC caused no biofilm formation but yeast-to-hyphal or pseudo-hyphal morphogenesis in both clades. Clade III showed a superior capacity in reducing membrane permeability to the drug through chemically tailoring chitin structure with a high degree of acetylation and fatty acids networks with significantly elongated chains. This study shows the suitability of the *in situ* Raman method in characterizing susceptibility and stress response of different *C. auris* clades to antifungal drugs, thus opening a path to identifying novel clinical solutions counteracting the spread of these alarming pathogens.

## Introduction

Invasive candidiasis associated with biofilm formation is a fungal infection of difficult therapeutic management, further complicated by the rise of species with strong resistance against antifungal drugs (Sato et al., 2009; Cavalheiro and Teixeira, 2018; Pappas et al., 2018). Fungal colonization contributes to lower lung function and might lead to severe lung disease (Sato et al., 2009). Since different fungal species differ in drug resistance characteristics (Ademe and Girma, 2020; Dahiya et al., 2020), prompt diagnosis of *Candida* species and accurate susceptibility monitoring of antifungal resistance are key diagnostic essentials.

Among *Candida* species, the multi-drug resistant pathogenic *Candida auris* (*C. auris*) (Sato et al., 2009) can cause nosocomial infections in immunocompromised persons. The presence of this pathogen is often associated with consistent candidemia and high mortality rate, thus representing a serious threat to global health (Marak and Dhanashree, 2018; Ademe and Girma, 2020; Dahiya et al., 2020; Du et al., 2020) ^1^. *C. auris* genotypes, which specialize to particular geological areas, frequently exhibit multidrug-resistance with specific clades displaying unusual sensitivity profile. *C. auris* is comprehensive of five distinct clades, classified according to where the first isolate of the clade was reported: South Asian (Clade I), East Asian (Clade II), African (Clade III), South American (Clade IV), and Iranian (Clade V) (Chow et al., 2019; Welsh et al., 2019). These clades possess specific genetic and biochemical characteristics, which link to their response to different drugs (Forsberg et al., 2019; Guadalupe Frias-De and Martinez-Herrera, 2020). It should be noted that misidentifications and inconsistent results from commercialized kits often complicate candidiasis management, lead to treatment failure, and potentially amplify multidrug-resistance risks (Snayd et al., 2018). Given the progressively recorded spread of *C. auris* into an increasing number of countries, measures of preventive control, and deeper knowledge of physiological responses to specific drugs are paramount to counteract lethal effects and to interrupt transmission.

In a previous paper (Pezzotti et al., 2021), we demonstrated the power of Raman spectroscopy and imaging in studying *Candida* species. Empowered with a specifically tailored machine-learning algorithm, Raman spectroscopy could enable swift identification of *C. auris* clades with respect to *Candida albicans* ones. High-resolution Raman hyperspectral imaging was also applied as a molecular microscopy tool for *in situ* label-free screening of living *Candida* yeast cells. Besides concurrently accomplishing accuracy and testing speed, the Raman imaging approach showed potential for revealing fundamental details related to structure, virulence, and drug resistance characteristics of different *Candida* species.

In this paper, we build upon our previous Raman analyses of living *Candida* cells and monitor *in situ* the susceptibility of *C. auris* Clades II and III to two primary antifungal compounds: Amphotericin B and 5-flucytosine. These two clades were purposely selected. Clade II, which was first discovered in Japan (Sato et al., 2009), has a propensity for infecting the ear, while Clade III causes invasive infections and large scale outbreaks (Welsh et al., 2019). Regarding drug resistance, Clade III has been observed to be resistant to multiple antifungal drugs including polyenes, while Clade II has not yet been reported to possess drug resistance (Forsberg et al., 2019). Raman imaging of *C. auris* clades before and after drug treatment gives us a chance of delving into the metabolic responses of different *Candida* species and molecular-scale candidacidal mechanisms behind different antifungal compounds, thus refining the state-of-art understanding of their distinct actions, and detailing the structural modifications of this dangerous pathogen in response to different drugs.

## Experimental Procedures

### *Candida* Clades and Immunochemistry Experiments

Two *C. auris* clades were provided by Teikyo University, as follows: the LSEM 3673 (belonging to the South African Clade III), and the LSEM 0643 (or JCM15448T; belonging to the East Asian Clade II). The *C. auris* clades were cultured in brain heart infusion (BHI) broth (NISSUI PHARMACEUTICAL Co. Ltd., Tokyo, Japan) at 36°C for 24 h under atmospheric pressure. The yeast Clade II and III were then treated for 24 h with 2.50 μg/ml Amphotericin B (AmB; FUJIFILM Wako Pure Chemical Corporation, Osaka, Japan) or 2 mg/ml 5-flucytosine (5-FC; Tokyo Chemical Industry Co., Ltd., Tokyo, Japan) at 36°C under atmospheric pressure, and then characterized by immunochemistry assays and Raman spectroscopy in comparison with untreated samples.

The metabolic activity of the *C. auris* was characterized by WST colorimetric assay (Microbial Viability Assay Kit-WST, Dojindo, Kumamoto, Japan). The kit employed the WST-8 indicator producing a water-soluble formazan dye upon reduction mediated by electrons. The amount of formazan dye directly correlated with the number of live microorganisms. Solutions were then analyzed using microplate readers (EMax, Molecular Devices, Sunnyvale, CA, USA) and cell viability estimated from calibrated optical density values.

The Periodic Acid Schiff (PAS) staining method was also utilized for cell visualization. In this test, the cells were fixed with 4% paraformaldehyde (PFA), washed with distilled water, and incubated with 0.5% Periodic Acid Solution (Wako Pure Chemical Corporation, Osaka, Japan) for 10 min. Staining with Schiff's Reagent solution (Wako Pure Chemical Corporation) was then performed for 15 min. After successively washing with sulfurous acid solution and water, the cells were observed under a fluorescence microscope (BZ-X700; Keyence Co., Osaka, Japan).

In an additional cell-imaging procedure, cells were washed with phosphate-buffered saline, fixed with 4% PFA for 10 min at room temperature, and washed again in wash buffer concentrate for three times (5 min each). Successively stained for 60 min in the dark using a specific fluorescent kit for cholesterol (Filipin III blue, Cayman Chemical, Ann Arbor, MI, USA) with dilution 1:100 and washed again with wash buffer concentrate, cells were imaged under a fluorescence microscope (BZX710; Keyence Co., Osaka, Japan).

### *In situ* Raman Spectroscopy

Raman spectra were collected *in situ* on control (i.e., drug-unexposed) and drug-exposed *C. auris* Clades II and III living yeast cells. A dedicated instrument (LabRAM HR800, Horiba/Jobin-Yvon, Kyoto, Japan) was used, which focused the incoming laser and collected the Raman light through a 20× optical lens. Set in confocal mode, the spectroscope was equipped with a holographic notch filter enabling efficient and high spectrally resolved acquisitions. The excitation wavelength was 532 nm operating with a power of 10 mW. A spectral resolution of better than 1 cm^−1^ was achieved by using an internal reference (neon emission) to calibrate the spectrometer. The Raman scattered light was monitored by a single monochromator connected with an air-cooled charge-coupled device (CCD) detector (Andor DV420-OE322; 1,024 × 256 pixels). The acquisition time for a single spectrum was typically 10 s. Thirty spectra were collected at different locations over an area of ~2 mm (Pappas et al., 2018) for each clade and averaged in order to obtain an average spectrum for each clade and each treatment.

Spectral deconvolution was performed by means of a machine-learning matching algorithm linked to a Raman spectra library of pure compounds (Pezzotti et al., 2021). The library contained more than 40 elementary compounds, including polysaccharides (e.g., chitin, β-1,3-glucans, β-1,6-glucans, α-1, 3–glucans), mono- and disaccharides (e.g., trehalose, β-D-glucose, D-dextrose), lipids (e.g., triolein, trilinolein, 1,2-dipalmitoyl-L-α-lecithin), polyols (e.g., D-(+)-Arabitol and L-(-)-Arabitol), and other key molecules such as adenine, ergosterol, and glycine. A library of spectra from pure compounds was available, which was constructed by collecting data with a highly resolved spectrometer (T-64000; Jobin-Ivon/Horiba Group, Kyoto, Japan) equipped with a nitrogen-cooled charge-coupled device detector (CCD-3500V, Jobin-Ivon/Horiba Group, Kyoto, Japan). The excitation source in these latter experiments was a 514 nm line of an Ar-ion laser operating with a nominal power of 200 mW. The spectral resolution was better than 1 cm^−1^.

Raman imaging of *C. auris* cells was conducted in a dedicated Raman device (RAMANtouch, Nanophoton Co., Minoo, Osaka, Japan) operating in microscopic measurement mode with confocal imaging capability in two dimensions. The RAMANtouch microscope, which was specifically designed to be compatible with cells' life, is capable of ultra-fast simultaneous acquisition of a line of 400 spectra. It applies an excitation source of 532 nm with a spectral resolution of ~2 cm^−1^ (spectral pixel resolution equal to 0.3 cm^−1^/pixel). Its accuracy in laser spot location is 100 nm. Raman hyperspectral images could then be generated from the raw spectra using commercially available software (Raman Viewer, Nanophoton Co., Minoo, Osaka, Japan). Raman images were generated using intensity ratios in the normalized spectra and using an average intensity of ±3 pixels around the band nominal location.

### Spectral Treatments and Deconvolution

Experimental Raman spectra were treated with a baseline subtraction procedure and then automatically deconvoluted into a series of Lorentzian/Gaussian sub-bands. Baseline subtraction was first performed according to a polynomial fitting procedure, followed by spectral normalization to the glucose ring signal at 478 cm^−1^ and successive spectral averaging to obtain representative spectra for each clade sample investigated. All these procedures employed options available in commercial software (LabSpec 4.02, Horiba/Jobin-Yvon, Kyoto, Japan).

Average spectra, S_av_(ν), were then fitted to an automatic solver, which exploited a linear polynomial expression of Voigtian functions, V(Δν, σ, γ); with ν, Δν, σ, and γ representing the Raman frequency, the shift in frequency from each sub-band's maximum (ν_0_), the standard deviation of each Gaussian component, and the full-width at half-maximum of the Lorentzian component, respectively. Best matching to the average spectrum was searched for as the one giving the minimum value in the following equation:


(1) $$\begin{array}{l} S_{\text{av}}\left(\nu \right)-\Sigma_{\text{i}}\alpha_{\text{i}}\Sigma_{\text{j}}\beta_{\text{ij}}V_{\text{ij}}\left(\nu_{0},\Delta \nu ,\sigma ,\gamma \right)≅0 \end{array}$$

where, the index i locates each compound in a series of n contributing to the overall spectrum, and the index j locates the Lorentzian/Gaussian sub-bands of a series of m in the Raman spectrum of each compound of an n series. An in-house built computer program located selected sub-bands upon by picking them up from those of pre-selected compounds belonging to the above-mentioned library and according to previously published literature on the cellular structure of *Candida* species. The algorithm in Eq. (1) then located the best fit to the experimental spectra. The criteria selected for the computational procedure were, as follows: (i) preserving relative intensities (β_ij_) in pure compound spectra, (ii) fixing spectral positions (ν_0_), and (iii) fixing full-width at half-maximum (σ and γ) values for individual sub-bands of the deconvoluted spectra from each elementary compound while allowing fluctuations of ±3 cm^−1^ for both band position and width to take into account the resolution of the spectrometer and the possibility of slight alterations in molecular structure. The above three criteria provide a series of mathematical constraints leading to univocal deconvolution of the experimental spectra. When the solver failed to fit specific spectral locations according to the pre-selected library, a new compound was searched for in the library to be added to the pre-selected compounds. Then, the overall intensity contribution (α_i_) of each elementary compound was newly adjusted to locate a best fit for the experimental spectrum.

### Statistical Analysis

The statistical relevance of the experimental data was analyzed by computing mean values and standard deviations. Their statistical validity was evaluated by applying the unpaired Student's t-test (Guo and Yuan, 2017). In each sample size of *n* = 4, *p* < 0.005 and < 0.001 were considered statistically significant and labeled with one and two asterisks, respectively.

## Results

### Immunochemical Response of Untreated and Drug-Treated Clades

**Figure 1:**
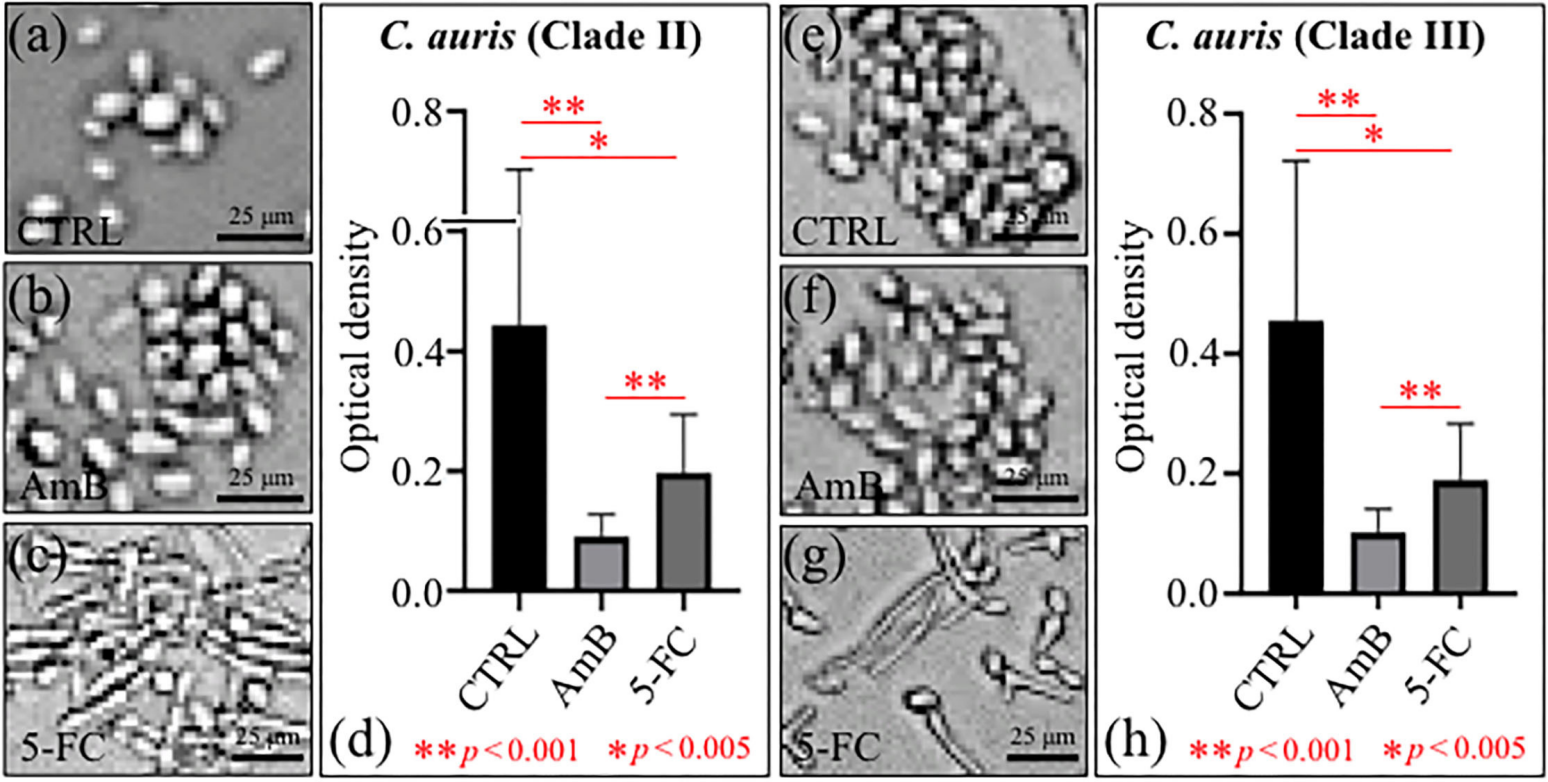
Optical micrographs of *C. auris* cultures before (CTRL label) and after 24 h treatment with different drugs (cf. labels): Clade II **(a–c)** and Clade III **(e–g)**; OD plots obtained from microbial viability assays are given in **(d,h)** for Clades II and III, respectively.

The levels of proliferation of *C. auris* yeast cells after 24 h treatment with different drugs are summarized in Figures 1a–d Clade II and Figures 1e–h Clade III (cf. labels in inset). Quantification was made using optical density (OD) values from microbial viability assays (cf. plots in Figures 1d,h for Clades II and III, respectively). Statistically significant differences in the viability plots proved the candidacidal efficacy of both drugs. However, the highest drug efficacy was found for AmB in both clades. An important feature that could be noticed from comparing optical micrographs before and after drug treatments was a clear filamentous growth into hyphae in the case of 5-FC treatment. This characteristic was common to both Clades II and III (cf. Figures 1c,g, respectively).

**Figure 2:**
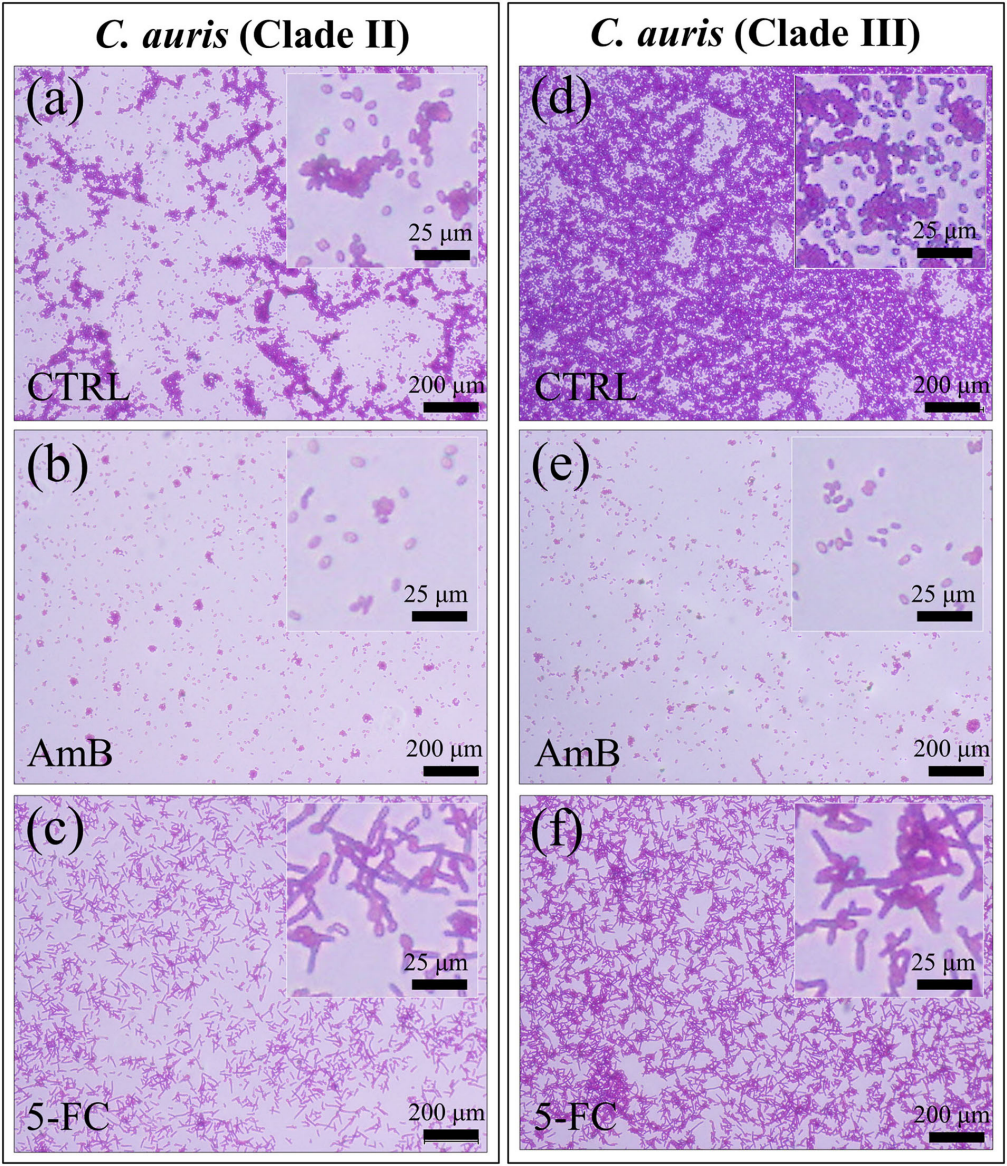
Fluorescence micrographs of violet PAS-stained *C. auris* cultures before (CTRL label) and after 24 h treatment with different drugs (cf. labels in inset): Clade II **(a–c)** and Clade III **(d–f)**. The micrographs stain polysaccharides in violet color. Note the most significant reduction in cell density upon AmB treatment [cf. **(b,e)**] for Clades II and III, respectively) and the hyphal morphogenesis in the enlarged images in inset to **(c,f)**.

Micrographs are shown in Figures 2a–f for PAS-stained Clades II and III, respectively. The micrographs, which stain polysaccharides in violet color, confirmed the state of proliferation observed in Figure 1 after 24 h drug exposure, with the most significant reduction in cell density being detected upon AmB treatment (cf. Figures 2b,e for Clades II and III, respectively). Enlarged images of stained cells are given in insets to each section of Figure 2. In (c) and (f), the *C. auris* detector kit provided a clear visual indicator of hyphal morphogenesis upon 5-FC treatment, independent of clade. Of note, a direct cell counting showed a ~4-log_10_ reduction in pathogen viability (99.99%) upon AmB treatment in both clades. A closer look to enlarged images (in inset to Figures 2c,f) revealed hyphae with slightly longer filament length in Clade III, while the majority of cells in Clade II appeared to take the morphology of pseudo-hyphae.

**Figure 3:**
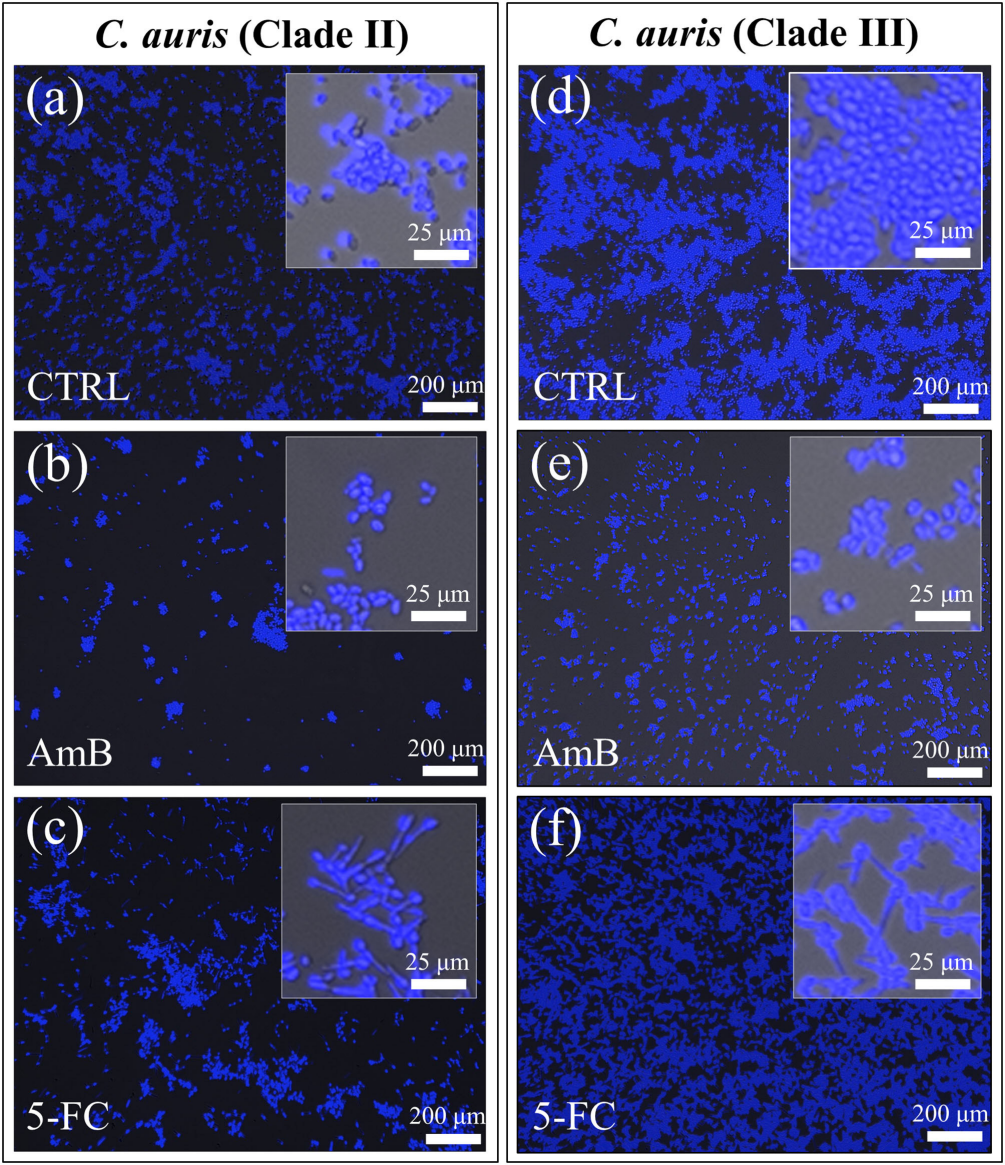
Confocal laser micrographs of violet Filipin blue-stained *C. auris* cultures before (CTRL label) and after 24 h treatment with different drugs (cf. labels in inset): Clade II **(a–c)** and Clade III **(d–f)**. The micrographs stain ergosterol in blue color. Filipin blue outputs were in line with the PAS violet ones (cf. Fig. 2) showing the most significant reduction in cell density upon AmB treatment [cf. **(b,e)** for Clades II and III, respectively] and the occurrence of hyphal morphogenesis in 5-FC treated cultures of both clades [cf. enlarged images in inset to **(c,f)**].

Figure 3 shows confocal laser microscopy results on as-cultured control yeast cells, and cells exposed for 24 h to both AmB and 5-FC treatment (cf. labels). Before observation, samples were treated with fluorescent Filipin blue stain, which specifically links to membrane ergosterol molecules. Quantitative data based on pixel counting (not shown) gave results consistent with those obtained by OD and violet staining (in Figures 1, 2, respectively). Enlarged areas shown in inset to each micrograph again clearly revealed that hyphal morphogenesis only occurred in the 5-FC-treated samples. As mentioned before, the Filipin blue stain directly links to ergosterol, which is an important plasma membrane lipid in *Candida* species and regulates its fluidity, permeability, and integrity. Enrichment in ergosterol has been associated with both susceptibility and reactions of *Candida* species to a variety of stresses, such as ionic, osmotic, oxidative pressure, and treatment by antifungal drugs (Rodrigues, 2018). However, the present Filipin-blue staining could not reveal any clear difference neither in the tone of blue nor in its location as recorded on fluorescence micrographs taken before and after treatments with different drugs. The only morphological hint to a reaction of the *C. auris* cells of both clades to a stress source was their conversion into filamentous morphologies upon exposure to the 5-FC drug. Hyphal differentiation in *Candida* species has indeed been correlated to subtoxic oxidative stress (Nasution et al., 2008), although their ability to adapt to the host's blood, including resistance to oxidative stress, proved independent of hyphal length (Maza et al., 2017). Raman spectroscopic characterizations were then conducted to confirm at the molecular scale the physiological reactions of different *C. auris* clades to AmB and 5-FC treatments, as described hereafter.

### Average Raman Spectra of *C. Auris* Before and After Drug Treatments

**Figure 4:**
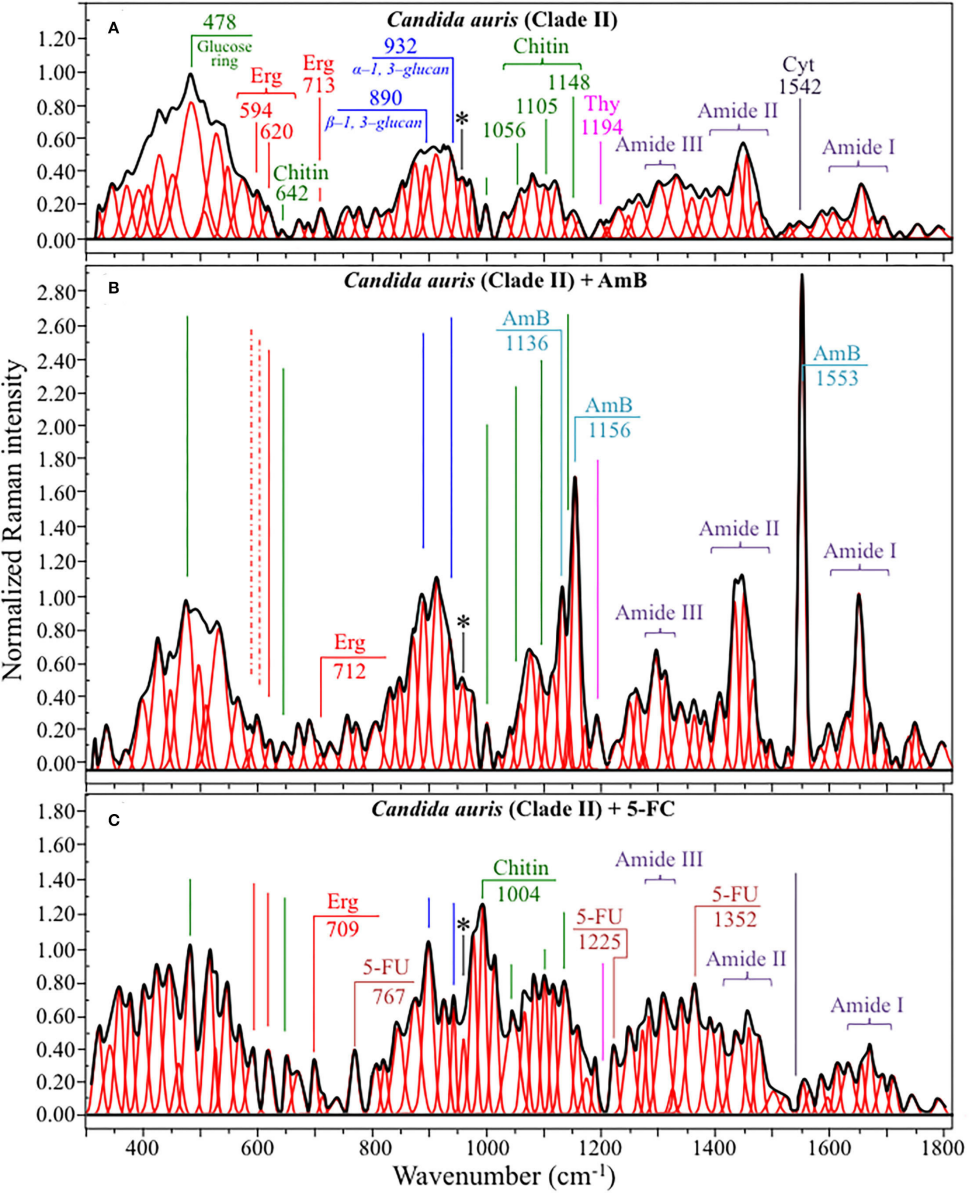
Average Raman spectra of *C. auris* Clades II in the wavenumber interval 300~1,800 cm^−1^; sections **(A–C)** correspond to as cultured cells, and cells treated with AmB and 5-FC, respectively. Spectra are normalized to the glucose ring band at 478 cm^−1^ and deconvoluted into Lorentzian/Gaussian sub-bands by means of the machine-learning algorithm described in Section Spectral Treatments and Deconvolution. The asterisk locates the C-CH_3_ deformation band at 956 cm^−1^.

**Figure 5:**
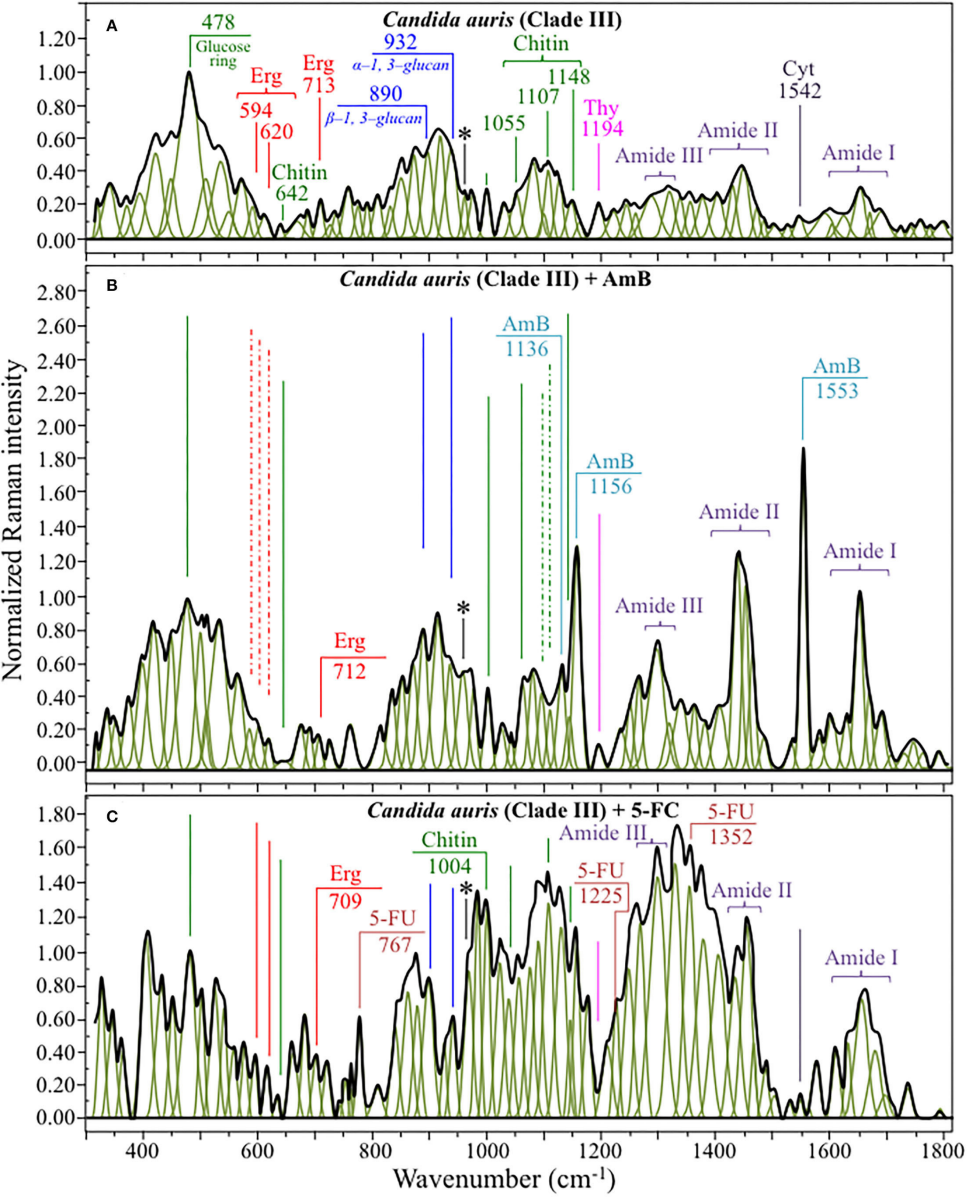
Average Raman spectra of *C. auris* Clades III in the wavenumber interval 300~1,800 cm^−1^; sections **(A–C)** correspond to as cultured cells, and cells treated with AmB and 5-FC, respectively. Spectra are normalized to the glucose ring band at 478 cm^−1^ and deconvoluted into Lorentzian/Gaussian sub-bands by means of the machine-learning algorithm described in Section Spectral Treatments and Deconvolution. The asterisk locates the C-CH_3_ deformation band at 956 cm^−1^.

Figures 4, 5 show average Raman spectra of *C. auris* Clades II and III, respectively, as collected in the wavenumber interval 300~1,800 cm^−1^. In sections (a), (b), and (c) of each figure, spectra correspond to as-cultured cells, and cells treated with AmB and 5-FC, respectively. All spectra are shown after normalization to the glucose ring band at 478 cm^−1^ and deconvolution into a series of Lorentzian/Gaussian sub-bands by means of the machine-learning algorithm described in Section Spectral Treatments and Deconvolution. General descriptions of the spectral bands for Clades II and III (in Figures 4, 5, respectively) have been given in a previous study (Pezzotti et al., 2021). The salient points of the Raman analyses of as-cultured and drug-treated cells are detailed in the following sections. In applying the machine-learning algorithm, the initial choice of molecular components included: β-1, 3–glucans, α-1, 3–glucans, chitin, ergosterol, D-arabitol, trehalose, adenosine, guanosine, cytidine, and thymidine. Statistical (chemometric) validations of the shown average spectra and a comparison between spectra from as-cultured Clades II and III were given and discussed in Pezzotti et al., 2021. Average spectra from as-cultured clades showed a quite high degree of similarity. Principal component analysis confirmed that it is not statistically possible to distinguish between the Raman spectra of the two presented *C. auris* clades (Pezzotti et al., 2021).

### Selection of Molecular Markers in the Raman Spectrum of *C. Auris*

**Figure 6:**
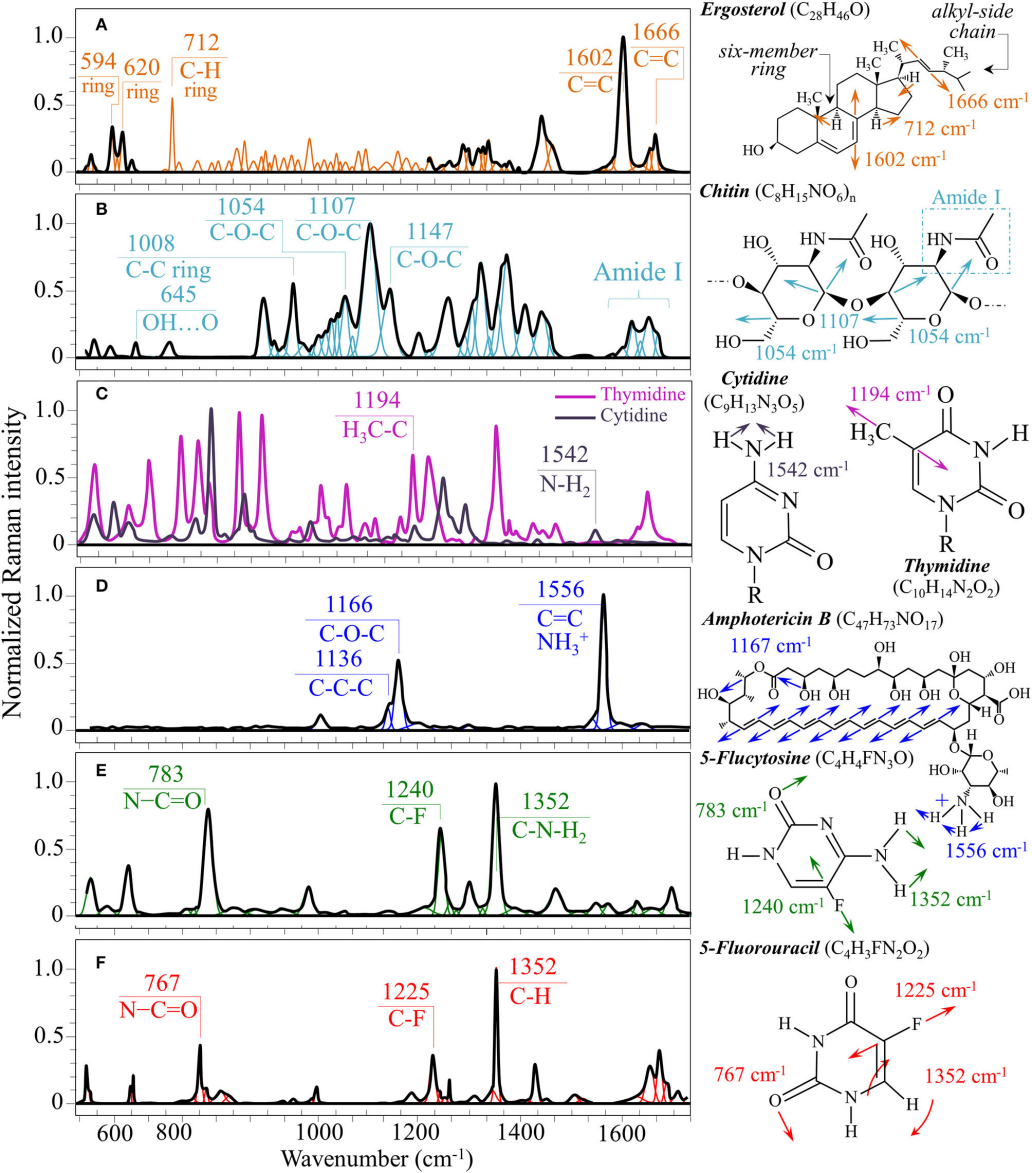
Structures, main vibrational modes, and reference Raman spectra of **(A)** ergosterol, **(B)** chitin, **(C)** cytidine/thymidine, **(D)** AmB, **(E)** 5-FU, and **(F)** 5-FU pure compounds.

Structures and reference Raman spectra of ergosterol, chitin, and cytidine/thymidine pure compounds are given in Figures 6A–C, respectively. The spectra were taken from a library of Raman spectra collected on elementary molecules. The machine-learning algorithm in Eq. (1) located two low-frequency bands (at 594 and 620 cm^−1^), both related to ring vibrations, as mainly (>95%) contributed by steroid ergosterol (cf. spectrum of pure ergosterol in Figure 6A together with selected vibrational markers) (Zivanovic et al., 2018). On the other hand, the main C=C stretching bands at 1,602 and 1,666 cm^−1^ could not serve as ergosterol markers in *C. auris* spectra because of their strong overlap with signals from other biomolecules (e.g., amino acid residues and DNA bases). The relatively intense and isolate band at ~713 cm^−1^ was contributed by ergosterol (cumulative of C-H bending and ring deformation) (Zivanovic et al., 2018), but it also contained non-negligible contributions from D-arabitol, chitin, and adenine. Upon comparing the sum of the relative intensities of the 594/620 cm^−1^ doublet in the spectra of different *C. auris* clades (cf. Figures 4A, 5A), the fraction of ergosterol appeared to be higher in Clade II (0.50) as compared to Clade III (0.34). We anticipate that the trend of ergosterol bands is important because its depletion or enhancement, and its structural alteration reflect the response of *C. auris* clades to different drugs with impact on their antifungal resistance (Kean and Ramage, 2019). This point will be discussed later in more details.

As discussed in a previous report on the same *C. auris* clades studied here (Pezzotti et al., 2021), machine-learning examination of band contributions from biomolecules included in the yeast structure indicated the sub-band at ~890 cm^−1^ as marker for β-1, 3–glucans, while the antisymmetric ring vibration at 932 cm^−1^ was a fingerprint for α-1, 3–glucans (cf. Figures 4A, 5A for Clade II and III, respectively). The above choice of the 932 cm^−1^ marker was in line with previously published studies of α-1, 3–glucans (Synytsya et al., 2009; Mikkelsen et al., 2010). The fractional ratio of α- to β-glucans of the yeast wall structure, calculated as the intensity ratio of the 932 to the 890 cm^−1^ bands, was very similar in both clades (i.e., 1.09 and 1.03 for Clades II and III, respectively).

A spectral region representative of C-C-C and C-O-C stretching in polysaccharides corresponds to the wavenumber interval between 950 and 1,200 cm^−1^. Despite structural similarities, giving almost identical spectroscopic features, distinctions among different polysaccharides can yet be made upon using bands in this peculiar region. In the case of chitin (Figure 6B), besides C-O-C bonds between rings, another main structural difference resides in the presence of an amide group (to replace an OH group) as a structural sub-unit in the polymeric chain (Gussem et al., 2005). Structural peculiarities confer distinctive vibrational characteristics as compared to other polysaccharides (cf. Figure 6B). The triplet at 1,054, 1,107, and 1,147 cm^−1^ and the low-frequency band at 645 cm^−1^ stands conspicuously free from overlap with other signals in the spectrum of *C. auris*, and thus could serve as fingerprints for chitin. Other bands (e.g., the one at ~1,004 cm^−1^ from C-C ring breathing) are contributed by chitin, but also non-negligibly by other polysaccharides (Gussem et al., 2005); therefore, they cannot univocally be selected as markers for the chitin molecule. Upon comparing the relative intensities of the above chitin-marker bands (i.e., with respect to the 478 cm^−1^ glucose ring band) between as-cultured clades, one finds that Clade III is ~20% richer in chitin as compared to Clade II. Since the chitin structure adds rigidity and structural support to the yeast cell walls, Raman data prove that Clade II possesses a membrane with higher flexibility.

An important detail regarding chitin-related bands lies in the distinction between signals from C-O-C groups within and between rings. The ether C-O-C band between rings is seen at 1,107 cm^−1^, while C-O-C bonds within rings scatter at 1,054 cm^−1^ (cf. Figures 4A, 5A). The band intensity ratio, I_1107_/I_1054_, can thus be taken to represent the ratio between ether and ring C-O-C bonds in chitin, the higher the ratio the longer the chitin chains. As-cultured Clade III showed an I_1107_/I_1054_ ratio 25% higher than Clade II (~1.5 vs. 1.2); thus, its chitin structure incorporated longer chains. Degradation of the chitin structure, namely, the decomposition of oxygen bridges between rings is expected to display as a lowering of the ether C-O-C signal at 1,107 cm^−1^ with respect to that of ring C-O-C bonds at 1,054 cm^−1^ (labeled with an asterisk in Figures 4, 5).

Amides I, II, and III signals (i.e., in the wavenumber intervals 1,600~1,700 cm^−1^, 1,400~1,500 cm^−1^, and at around 1,300 cm^−1^, respectively) are peculiar to the N-acetyl group of chitin (cf. labels Figures 4A, 5A). The intensities of these signals are thus related to levels of acetylation/deacetylation in the membrane structure of *Candida* clades. However, there were no significant differences regarding amide signals between as-cultured Clades II and III. Additional discussions on amide bands will be given later in the context of clade response to drug treatments. Finally, note that chitin deacetylation might induce variations in the I_1107_/I_1054_ chitin ratio, the higher the degree of deacetylation the lower the ratio (Binias et al., 2016).

We also looked into the possibility to resolve individual band components from DNA and RNA. Unfortunately, the majority of prominent bands from DNA nucleosides appeared in the low-wavenumber interval below 900 cm^−1^ and could not be univocally assigned to individual nucleosides because of a strong overlap with signals from other biomolecules. However, machine-learning examination indicated a relatively strong sub-band at ~1,194 cm^−1^ (C-CH_3_ stretching) (D'Amico et al., 2015) and a minor band at 1,542 cm^−1^ (NH_2_ scissoring) (Barboza et al., 2017) as mainly contributed (>95%) by thymidine and cytidine nucleosides, respectively (cf. spectra of elementary molecules and related Raman markers in Figure 6C). These bands were then selected as markers for these two nucleosides (cf. labels in Figures 4, 5).

**Figure 7:**
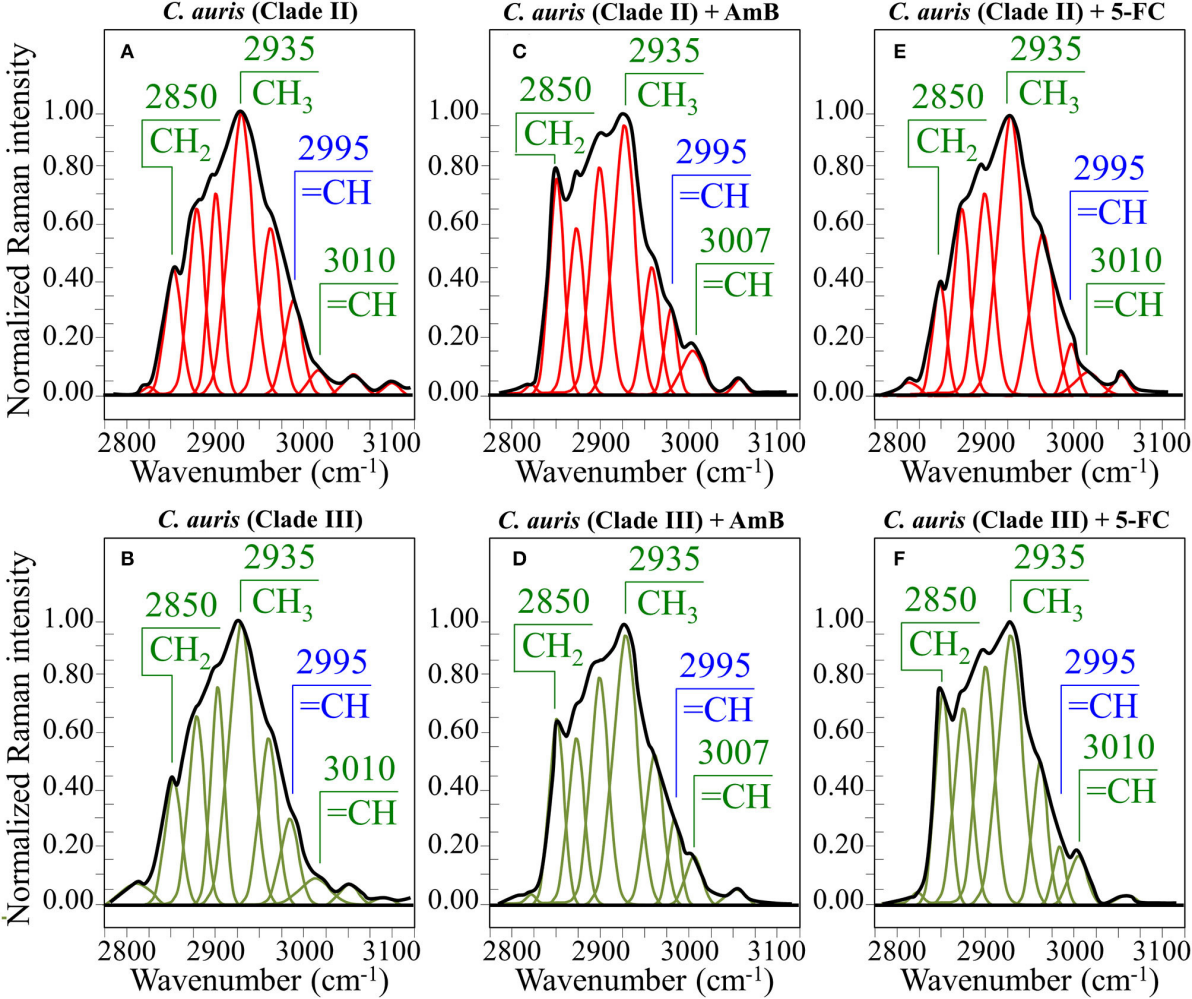
Average Raman spectra in the spectral interval 2,800~3,100 cm^−1^ for Clade II/Clade III as-cultured cells **(A,B)**, cells treated with AmB **(C,D)**, and 5-FC **(E,F)**. Spectra are normalized to the CH_3_ band at 2,935 cm^−1^ and deconvoluted into Lorentzian/Gaussian sub-bands.

In an attempt to search for additional Raman markers, spectra were also collected in the high-frequency zone between 2,800 and 3,100 cm^−1^ (cf. spectra normalized to the strongest band at 2,935 cm^−1^ in Figures 7A,B for Clades II and III, respectively). It should be noted at the outset that it is difficult to assign peaks in this region to specific chemical groups. However, the predominant vibrational contributions of CH_3_, CH_2_, and CH groups can be distinguished, which gives hints for identifying structural features in specific structural context, as discussed later. Deconvolution into Lorentzian-Gaussian sub-bands in the high-frequency zone was made according to Borchman et al. (1999) and Kolijenovic et al. (2005). This spectral zone is basically comprehensive of eight sub-bands: at 2,820 cm^−1^ (CH_2_ symmetric stretching in fatty lipids) at ~2,850 cm^−1^ (CH_2_ symmetric stretching in fatty acids) and its Fermi resonance at ~2,886 cm^−1^, at 2,900 cm^−1^ (CH stretching), at ~2,935 cm^−1^ (chain-end CH_3_ symmetric stretching), at ~2,960 cm^−1^ (out-of-plane chain end and anti-symmetric CH_3_ stretching), at 2,995 cm^−1^ (=CH stretching), and at 3,010 cm^−1^ (unsaturated =CH stretching). Two additional weak bands could be seen at ~3,056 and 3,100 cm^−1^, which can be assigned to OH and NH stretching vibrations. Small fluctuations in frequency for the above band components might arise from differences in the molecular structures in which the bonds are comprised. In the zone 2,800~3,100 cm^−1^, the deconvoluted sub-bands in the spectra of Clades II and III before exposure to drugs appeared very similar (cf. Figures 7A,B).

### Structures and Raman Spectra of AmB and 5-FC Drug Compounds

AmB and 5-FC represent two main treatments in current therapies for invasive candidiasis (Vermes et al., 2000; Mesa-Arango et al., 2012); they can also be used in combined therapies to achieve synergistic effects in severe cases of cryptococcal pneumonia (Dromer et al., 2008). Despite its severe acute and chronic toxicities (Miller and Bates, 1969; Al-Dhaheri and Douglas, 2010; Grela et al., 2018), AmB is still considered the gold standard for severe mycoses treatment (Ostrosky-Zeichner et al., 2003; Lemke et al., 2005). On the other hand, 5-FC is one of the oldest antifungal drugs and has a wide spectrum of activity despite a reported drawback in monotherapy due to a rapid frequency resistance in cryptococci (Chang et al., 2021).

From a chemical point of view, AmB is a compound constructed with a hydrophobic polyene chain included in a macrolide ring closed by a lactone and linked to a mycosamine group. On the other hand, 5-FC is an organofluorine compound structurally similar to cytosine, but presenting a fluorine atom at ring position 5 (fluorinated pyrimidine). These peculiar structures reflect into intense specific features in the respective Raman spectra of these drugs. Figures 6D,E show structures, Raman spectra and principal vibrational modes of AmB and 5-FC pure compounds, respectively. AmB presents two main bands at 1,556 and 1,167 cm^−1^; the former band, which is the most intense in the AmB spectrum, is a cumulative signal from –${\text{NH}}_{3}^{+}$ symmetric bending and C=C stretching, while the latter and its low-frequency shoulder at 1,136 cm^−1^ can be assigned to stretching in C-O-C and C-C-C bonds, respectively (Gagos and Arczewska, 2012). On the other hand, 5-FC presents several distinctive Raman bands, the most intense being found at 1,352 cm^−1^ (C-N-H_2_ symmetric bending), 1,240 cm^−1^ (C-F stretching), and 783 cm^−1^ (N-C=O bending) (Gunasekaran et al., 2006).

### Alterations of Clades II and III Spectra Upon Drug Treatment

The most striking feature in the spectra of both Clades II and III treated for 24 h with AmB was the presence of the two main bands of the drug molecule at 1,553 cm^−1^ and 1,156 cm^−1^ with a sharp shoulder at 1,136 cm^−1^ (cf. Figures 4B, 5B). Note that the Raman bands of AmB found in the spectra of both clades cultures maintained the same relative intensities and band morphologies as those observed in the spectrum of the pure AmB compound (cf. Figure 6D). Conversely, in both clade samples treated with 5-FC, significant intensity increases were found at 767, 1,225, and 1,352 cm^−1^ (cf. Figures 4C, 5C). These bands, which arise from vibrational modes similar to those described for 5-FC, do not precisely match the wavenumbers of 5-FC, and could be assigned to another fluorinated pyrimidine, namely, 5-fluorouracil (5-FU; cf. 5-FC and 5-FU structures, spectra, and main vibrational modes in Figures 6E,F, respectively) (Pavel et al., 2005). The formation of 5-FU to replace the original 5-FC structure will be detailed later in this section.

**Figure 8:**
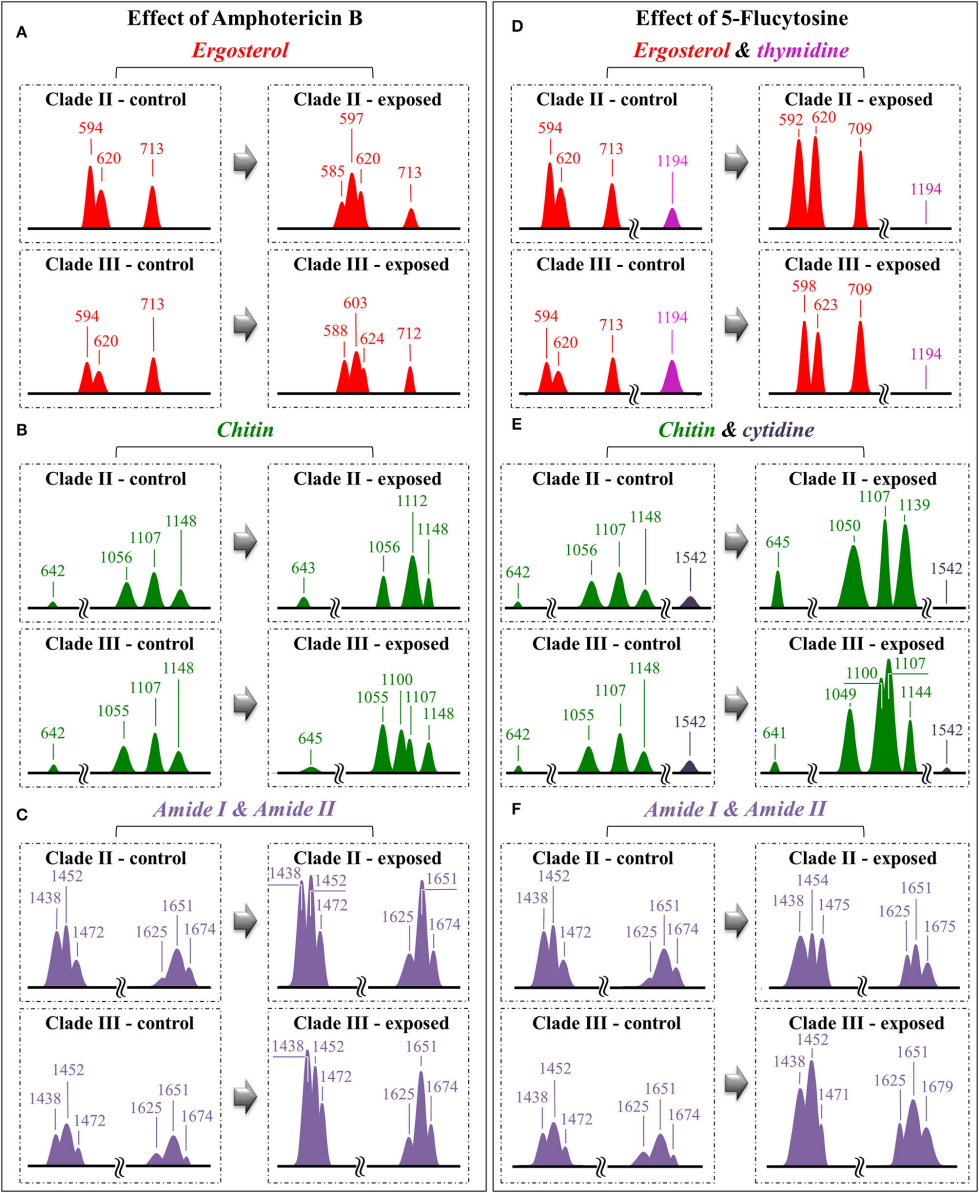
Diagrams drawn to summarize the spectroscopic differences recorded between contro and drug-treated *C. auris* (cf. labels); the sub-band components discussed in the text as markers of specific molecules are redrawn and compared with respect to their normalized intensities and wavenumber positions: for the effects of Amphotericin B, Ergosterol **(A)**, Chitin **(B)** and Amide I & II **(C)**; for the effect of 5-Flucytosine, Ergosterol & thymidine **(D)**, Chitin & cytidine **(E)** and Amide I & II **(F)**.

Regarding spectral characteristics other than those directly related to drug molecules, bold vibrational alterations could be found for both Clades II and III upon exposure to drugs in comparison with unexposed samples. These characteristics, which greatly differed for exposures to different drugs, are summarized hereafter. In order to help visualizing the spectroscopic findings, all the discussed sub-band components, markers of specific molecules, were redrawn and directly compared in Figure 8.

Raman spectra of Clades II and III exposed for 24 h to AmB (cf. Figures 4B, 5B, respectively):

- The relative intensity (with respect to glucose ring band at 478 cm^−1^) of the ergosterol-related band at 713 cm^−1^ showed a decreasing trend (54 and 20% for Clades II and III, respectively; cf. Figure 8A). However, this band is also contributed by biomolecules belonging to other lipids, polysaccharides, and DNA bases. Therefore, its decreasing trend cannot be taken as a marker for ergosterol only. As a matter of fact, no decreasing trend could be found for the other ergosterol band markers at 594 and 620 cm^−1^ (cf. Figure 8A) Clade II, which was initially richer by ~32% in ergosterol (cf. Section Selection of Molecular Markers in the Raman Spectrum of *C. auris*), showed a 620 cm^−1^ band with neither intensity reduction nor wavenumber shift upon AmB treatment. On the other hand, a slight increase and a shift by ~4 cm^−1^ toward higher frequencies was recorded for Clade III. An important feature for both clades was the split of the 594 cm^−1^ band into two sub-bands at lower and higher wavenumbers (cf. broken lines in Figures 4B, 5B, 8A). Band split could find its rationale in the so-called AmB/ergosterol binding effect (i.e., leading to membrane permeability) (Bolard et al., 2008), and in the altered expression of sterol biosynthesis genes upon AmB treatments (Munoz et al., 2018). This point will be further discussed in the forthcoming Section Raman Insights Into Yeast Responses to AmB Exposure.
- Upon treating Clade II with AmB, the intensity of all selected chitin Raman markers (at 642, 1,056, 1,107, and 1,148 cm^−1^) increased, with the band at 1,107 cm^−1^ becoming strongly intensified by ~61% and shifting to 1,112 cm^−1^ (cf. Figure 8B). A similar increase was seen in the spectrum of Clade III but only for the band centered at 1,055 cm^−1^. On the other hand, the band at 1,107 cm^−1^ preserved the same intensity, but became a shoulder of a stronger component at ~1,100 cm^−1^. Also, the band at ~1,004 cm^−1^, which was not selected as a marker by machine learning algorithm (but yet contained strong contributions from chitin), appeared strengthened in both clades upon exposure to AmB. However, the increasing trend of this latter band was less pronounced for Clade II (cf. Figures 4B, 5B). Regarding the shift of the 1,107 cm^−1^ band to 1,112 cm^−1^ observed in Clade II, it could originate from the elimination of acetyl groups replaced by NH_2_ groups (Schenzel and Fischer, 2001). The ratio between ether C-O-C bonds and C-O-C bonds within rings, I_1107_/I_1056_, increased by ~25% in Clade II, while it could not obviously be computed for Clade III due to split into two new components of the band originally centered at 1,007 cm^−1^. The increase of the ratio, I_1107_/I_1090_, in Clade II can be interpreted as a fingerprint for increased chitin chain length. These points will be discussed in detail in Section Raman Insights Into Yeast Responses to AmB Exposure.
- The main difference in the structure of chitin with respect to other polysaccharides resides in the presence of an amide group at carbon 2 to replace an OH group (cf. Figure 6B). Bands characteristic of the N-acetyl group have been reported at around 1,300 cm^−1^ (Amide III), 1,528 cm^−1^ (Amide II), and 1,621/1,656 cm^−1^ (Amide I) (Focher et al., 1992; Rinaudo, 2006; Zhang et al., 2012). In both clades, all amide bands increased [consistently with other chitin markers as described in (ii)] upon treatment with AmB, thus confirming that amide signals are mainly arising from chitin (cf. Figures 4B, 5B, 8C). The occurrence of a doublet for the Amide I, which we see at 1,625 and 1,650 cm^−1^ in both clades, has been attributed to the existence of two different types of hydrogen bonds in α-chitin crystals, namely, intermolecular (C=O^…^HN) and intramolecular (C=O^…^HO(C6); C=O^…^HN) hydrogen bonds (Minke and Blackwell, 1978; Focher et al., 1992). Additional Amide I band components, seen as shoulders at 1,615~1,620 and 1,687 cm^−1^, are believed to arise from different chitin secondary structures or defects in small crystalline chitin fibrils (Focher et al., 1992). Using the relative intensities of the Amide III (C-N stretching and N-H bending) or 956 cm^−1^ (C-CH_3_ deformation) bands (the latter labeled with an asterisk in Figures 4, 5), it is possible to estimate the degree of chitin acetylation, the higher the intensity the higher the fraction of acetylated chitin chains (Zhang et al., 2012). Moreover, as mentioned above, the Amide I doublet represent vibrations from C=O bonds linked by hydrogen bonds to either NH or C-OH groups (Minke and Blackwell, 1978). Therefore, the relative amount of hydrogen bonds linked to C=O groups is represented by the areas under the respective Amide I bands. Based on the above structural interpretation of amide-related vibrations, it becomes clear that the treatment with AmB enhanced the presence of chitin structures with a higher degree of acetylation as compared to untreated clades. However, while an almost constant intermolecular-to-intramolecular hydrogen bond ratio (0.34 vs.0.32) was preserved in Clade II, such ratio dropped by ~30% in Clade III upon drug exposure. An additional feature in the high-frequency zone was a clear increase in relative intensity of the C=O and O-C=O ester bands at ~1,730 and 1,750 cm^−1^, respectively, in both clades (cf. Figures 4, 5). Spectral characteristics related to chitin will be discussed in the forthcoming Section Raman Insights into Yeast Responses to AmB Exposure.
- The α/β ratio of glucan structures, as calculated from the intensity ratio of sub-bands at 932 cm^−1^ (from α-1, 3–glucans) and 890 cm^−1^ (from β-1, 3–glucans), changed from a common value of ~1 before AmB exposure to a common value of 0.75 in both clades after AmB exposure. Of note, it is also the strong increase relative intensity of both glucans bands in both AmB-doped clades. The increase in glucan contents and the relative enrichment in β-1, 3–glucans upon AmB exposure represents a major change in cell-wall structure and could be the result of a regulatory mechanism to compensate for an abnormal fluidity of the membrane due to the drug-induced structural modifications of ergosterol (Mesa-Arango et al., 2016). However, the evident formation of biofilm suggests that β-1, 3–glucans belong to an extracellular matrix network built by the cells to bind to AmB thus reducing the drug susceptibility of the clades (Nett et al., 2007).
- In the spectral zone 2,800~3,100 cm^−1^, the intensity ratio of the sub-band at 2,850 cm^−1^ to that at 2,935 cm^−1^ can be taken as a marker of the ratio of the groups CH_2_ to CH_3_, from which the degree of unsaturation of fatty acids could be predicted (Borchman et al., 1999; Jamieson et al., 2018). After exposure to AmB, increases by 1.7 and 1.5 times were observed for the intensity ratio 2,850/2,935 cm^−1^ for Clades II and III, respectively (cf. Figures 7C,D, respectively). This ratio represents the ratio between C-H(CH_2_) and C-H(CH_3_) stretching bands, which is directly proportional to the chain length of (saturated and unsaturated) fatty acids (Jamieson et al., 2018). Its increase could be interpreted as a metabolic reaction (common to both clades) against AmB. In addition, the intensity increase of the saturation sensitive peak at ~3,005 cm^−1^ (=C-H stretching in unsaturated fatty acids) with respect to the C-H(CH_2_) stretching peak at 2,850 cm^−1^ is in linear correlation with number of H-C= relative to number of CH_2_ groups for unsaturated fatty acids (Jamieson et al., 2018). A common value of ~0.22 was found for this ratio for both unexposed clades. This value remained conspicuously constant in AmB exposed Clade II, while it increased by ~23% in Clade III (cf. Figures 7C,D). Such increase, which directly represents an increase in the fraction of unsaturated fatty acids, could be interpreted as an attempt of the clade to increase its membrane fluidity to compensate for the disruptive effect of the drug.
  *Candida* species have developed many strategies (or survival factors), to deal with different drugs, which include modifications of plasma membrane composition by increasing the content of fatty acids (Vanegas et al., 2012). This is indeed what our present Raman data show. The Raman results support the hypothesis that yeast cells modify the content of lipids in order to prevent and counteract AmB-induced interdigitation of the plasma membrane. In other words, changes in plasma membrane composition of yeast cells in response to AmB toxicity could represent the final attempt of yeasts to increase membrane fluidity to compensate for the disruption caused by the drug.
- It was not possible to monitor the cytidine nucleoside marker band at 1,542 cm^−1^, because of overlap by the much stronger signal from AmB molecules. However, the thymidine band at 1,194 cm^−1^ could yet be detected after drug exposure. It showed a fivefold increase in Clade II but a 25% reduction in Clade III.

Raman spectra of Clades II and III exposed for 24 h to 5-FC (cf. Figures 4C, 5C, respectively):

- A significant increase could be noted for any of the marker bands of ergosterol in both clades, with no band split and negligible band shifts. Therefore, both clades similarly reacted to the effect of 5-FC by enhancing their relative and absolute content of ergosterol. In agreement with general notions on yeast membranes and similar to the effect of quaternary ammonium salts (Siau and Kerridge, 1999; Xu et al., 2017), the present Raman data suggest that ergosterol abundance, which is key in reducing membrane permeability, was one main factor in stress adaptation for both clades.
- All marker bands of chitin significantly increased in both clades, including the band at 1,004 cm^−1^ contributed by chitin and other polysaccharides. However, clear differences could be recorded between clades in the relative intensities of different marker bands before and after drug exposure. In Clade II, both bands at 1,056 and 1,107 cm^−1^ showed a twofold intensity increase after drug exposure, while the most remarkable increase (by a factor 4) was found in the band at 1,148 cm^−1^, which also experienced a 9 cm^−1^ shift toward lower wavenumbers. In Clade III, bands at 1,055, 1,107, and 1,148 cm^−1^ showed increased intensities by factors 2.4, 2.9, and 2.5, respectively. The band at 1,055 cm^−1^ showed a clear shift of ~6 cm^−1^ toward lower frequencies. Moreover, a strong band component was newly recorded at 1,100 cm^−1^, a characteristic similar to that observed upon exposure to AmB (cf. Figure 8B). These spectral characteristics point to both fractional and at structural changes in chitin, and represent fingerprints of the metabolic changes occurring in different clades in reaction to the effect of 5-FC. In Clade II, the I_1107_/I_1056_ ratio between ether and ring C-O-C bonds, which increased by ~14%, is fingerprint for an increased length in chitin chains. The appearance of a relatively strong signal at 1,100 cm^−1^ in Clade III instead reflects the formation of chitin allomorphs (discussed further in the forthcoming Section Discussion). Both the above characteristics were substantially the same as those respectively observed in clades exposed to AmB, suggesting the existence of specific metabolic patterns for each clade in response to stress.
- Minor variations in the relative intensities of bands belonging to both Amides I and II were observed in Clade II upon 5-FC treatment, while significant increases were recorded for the same vibrations in Clade III (with the maximum intensity increase of 140% observed for the Amide II band at 1,452 cm^−1^). Consistently, exposure to 5-FC led to a significant increase of the Amide III band (at ~1,300 cm^−1^) and the C-CH_3_ deformation band (at 956 cm^−1^; labeled with an asterisk in Figures 4, 5), but only in Clade III. Following the same reasoning given for interpreting the spectroscopic behavior of amide bands in clades treated with AmB, it appears that exposure to 5-FC enhanced the fraction of chitin structures with a high degree of acetylation only in Clade III. An analysis of the Amide I doublet at 1,625 (intermolecular C=O^…^HN chitin bonds) and 1,650 cm^−1^ (intramolecular C=O^…^HO(C6) and C=O^…^HN chitin bonds) revealed that both clades experienced a clear increase in the intermolecular-to-intramolecular hydrogen bond ratio upon drug exposure. A clear increase in relative intensity of the C=O ester band (at 1,745 cm^−1^) upon 5-FC treatment could only be recorded in Clade III (cf. Figures 4C, 5C).
- Similar to the case of treatment with AmB, the α/β ratio of glucan structures switched from the value of ~1 characteristic of both unexposed clades to a common value of 0.66 after 5-FC exposure. The enrichment in β-1, 3–glucans together with the increased content of chitin could yet be interpreted as a physiological reaction of both clades to drug exposure, in the attempt to stiffen the membrane. Note, however, that the observed change in glucan structure could also be partly related to yeast-to-hyphae morphogenesis (cf. Figures 1c,g for Clades II and III, respectively). Other authors reported an enhanced β-glucan content in hyphae when cells were treated with a sub-inhibitory concentration of caspofungin (Wheeler et al., 2008).
- In the spectral zone 2,800~3,100 cm^−1^, the intensity ratio of the sub-band at 2,850 cm^−1^ to that at 2,935 cm^−1^showed a slight decrease (~10%) in Clade II, while it increased abruptly by ~1.8 times in Clade III upon exposure to 5-FC (cf. Figures 7E,F, respectively). As stated in the previous section, this ratio represents the intensity of C-H(CH_2_) to C-H(CH_3_) stretching bands; therefore, it is directly related to the chain length of (saturated and unsaturated) fatty acids, the higher the ratio the longer the chain (Jamieson et al., 2018). Moreover, the ratio between the intensity of the saturation sensitive peak at ~3,005 cm^−1^ (=C-H stretching, peculiar to unsaturated fatty acids) and the C-H(CH_2_) stretching peak at 2,850 cm^−1^ is in linear correlation with number of H-C= relative to number of CH_2_ groups for unsaturated fatty acids (Jamieson et al., 2018). This ratio remained conspicuously unchanged for both 5-FC exposed clades with respect to unexposed clades (cf. Figures 7E,F), thus showing that the above-mentioned elongation in fatty acid chains for Clade III did not involve any correspondent increase in fatty acid content.
- The Raman markers of thymidine and cytidine nucleosides (at ~1,194 cm^−1^ and 1,542 cm^−1^, respectively) completely disappeared in Clade II and appeared significantly weakened in Clade III upon exposure to 5-FC (cf. labels in Figures 4C, 5C).

The spectroscopic characteristics obtained on average spectra for both clades/treatments were then subjected to confirmation by means of further characterizations based on Raman mapping and imaging, according to protocols described in previous works (Pezzotti, 2021; Pezzotti et al., 2021).

### Raman Imaging of *C. Auris* Metabolic Reactions to Drug

The data shown in the previous section revealed fundamental differences between Raman spectra collected on Clades II and III before and after drug treatments and for different drugs as well. The detected differences included cell wall lipids and polysaccharides, specifically ergosterol and chitin, respectively, as well as DNA thymidine and cytidine nucleosides. In order to confirm the above findings, Raman mapping was performed with micrometric spatial resolution in order to image compositional differences in Clades II and III before and after exposure to AmB and 5-FC. The total number of Raman spectra collected in each map/image per each clade/drug was in the order of 10^6^ over an area of ~10^3^ μm^2^. The acquisition of a large number of space-resolved Raman spectra enabled statistical validation of the findings obtained on the average spectra shown in Figures 4, 5, as discussed elsewhere (Pezzotti et al., 2021).

**Figure 9:**
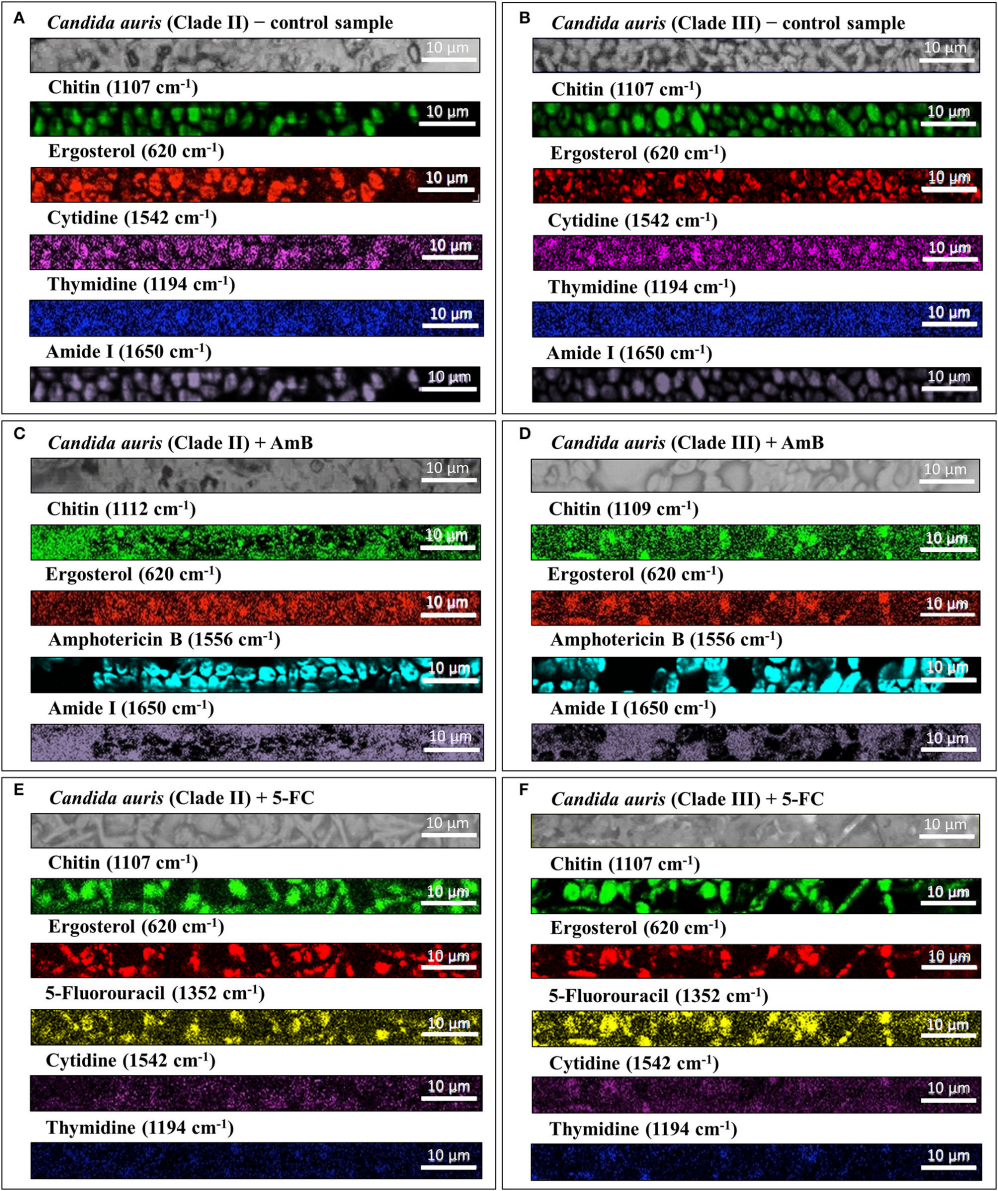
Optical micrographs and spatially resolved Raman data collected on different clades before and after treatments with different drugs; the latter are given as a series of hyperspectral maps for selected molecular markers: **(A,B)** control, **(C,D)** AmB-treated, and **(E,F)** 5-FC-treated Clades II and III, respectively; the targeted molecules (including those of the respective drugs) and the corresponding fingerprint wavenumbers are given by labels in inset to each section. The Raman mapping experiments were in good agreement with and helped to visualize the outputs of average spectral assessments summarized in Figure 8.

Figure 9 summarizes the findings of Raman mapping for different clades through a series of hyperspectral images for selected molecular markers. Sections (a) and (b), which refer to Clades II and III, respectively, shows (from top to bottom) optical micrographs of the cultures, and spatially resolved Raman maps (taken at fixed locations) of signals at 1,107 cm^−1^, 620 cm^−1^, 1,542 cm^−1^, 1,194 cm^−1^, and 1,650 cm^−1^ as representative markers for membrane chitin, ergosterol, DNA cytidine and thymidine nucleosides, and Amide I, respectively. Raman mapping experiments basically confirmed the trends observed in average spectra, namely, ~15% higher and ~20% lower fractions of ergosterol and chitin, respectively, in Clade II as compared to Clade III. On the other hand, no difference could be noticed in mapping nucleoside markers. In Section (c) and (d), optical images and Raman hyperspectral images of chitin, ergosterol, amphotericin B molecules, and Amide I (cf. monitored wavenumbers in inset) are shown (from top to bottom) for Clades II and III, respectively. As seen, AmB molecules, which were represented by the strong Raman marker at 1,556 cm^−1^, were clearly located inside cells for both clades, with the intense signals mainly localized around the cell membrane. On the other hand, signals from both chitin and Amide I (at 1,650 cm^−1^) were stronger and more spatially diffuse as compared to as-cultured cells for both Clades II and III. Also, the spatial distribution of ergosterol signals appeared more diffuse but similar in intensity to those of the as-cultured clades.

The effect of 5-FC treatment on Clades II and III is displayed in sections (e) and (f), respectively. Optical images, and maps of chitin, ergosterol, 5-FU, cytidine, and thymidine markers are given from top to bottom in each section. The penetration of the drug inside the cells is seen in both clades as a strong signal at 1,532 cm^−1^, which is the main marker for 5-FU (cf. Figure 6F). The chitin marker produced highly intense images in both clades, while maps for the chitin marker appeared to be substantially the same as those recorded for as-cultured cells. On the other hand, marker signals from both cytidine and thymidine nucleosides almost disappeared at any location for both clades. Trends in physiological responses of different clades to different drug treatments, as represented by Raman sub-bands of molecular markers, were averaged over the entire maps of Figure 9. As a general output, Raman mapping experiments conducted with microscopic spatial resolution matched with high precision the spectroscopic assessments made on larger areas with lower spatial resolution for both investigated clades (i.e., as summarized in Figure 8).

## Discussion

### Raman Insights Into Yeast Responses to AmB Exposure

Increased membrane permeability, which results from the formation of multimeric pores upon AmB to ergosterol binding, has been reported to be the main mechanism of action against *Candida* species (Brajtburg et al., 1990). However, data from a number of different research groups suggested that the effectiveness of AmB also depends on metabolic mechanisms, thus implying that an increased membrane permeability might not be the only mechanism responsible for the anticandidacidal action of the molecule (Phillips et al., 2003; Mousavi and Robson, 2004; Blum et al., 2008; Sharma et al., 2010). Perhaps, the most straightforward experimental proof in support of AmB possessing toxic mechanisms other than membrane pore formation was provided by Palacios et al. (2007). Those researchers made chemical modifications of the AmB molecule in order to fully compromise its ability to form membrane pores and yet recorded substantial fungicidal activity, thus providing strong evidence that pore formation is not essential or, at least, is not the only effective AmB mechanism of action. Other researchers pointed at oxidative stress as a powerful mechanism of action for AmB (Sokol-Anderson et al., 1986; Haido and Barreto-Bergter, 1989; Sangalli-Leite et al., 2011). Direct production of free radicals upon treatment with AmB has been visualized using fluorescence probes (Phillips et al., 2003; Sangalli-Leite et al., 2011). Lipid peroxidation, protein carbonylation, and apoptotic-like phenotypes have also been used as indirect indicators for the oxidative stress generated by AmB in fungal cells (Brajtburg et al., 1990; Siau and Kerridge, 1999; Palacios et al., 2007; Wheeler et al., 2008; Sharma et al., 2010; Xu et al., 2017). However, the molecular-scale mechanism by which AmB induces oxidative burst in *Candida* cells remains so far unknown. In particular, it is not clear whether the toxic radicals are generated by AmB autoxidation (Lamy-Freund et al., 1985; Sokol-Anderson et al., 1986), or if AmB simply induces the cells themselves to produce and accumulate reactive oxygen species through their mitochondrial activity (Mesa-Arango et al., 2014). Under physiological conditions, free radicals are produced in the mitochondria as natural by-products of the respiratory chain, but under conditions for which their endogenous concentration increases above a certain threshold, they produce structural alterations eventually leading to cell damage and death.

**Figure 10:**
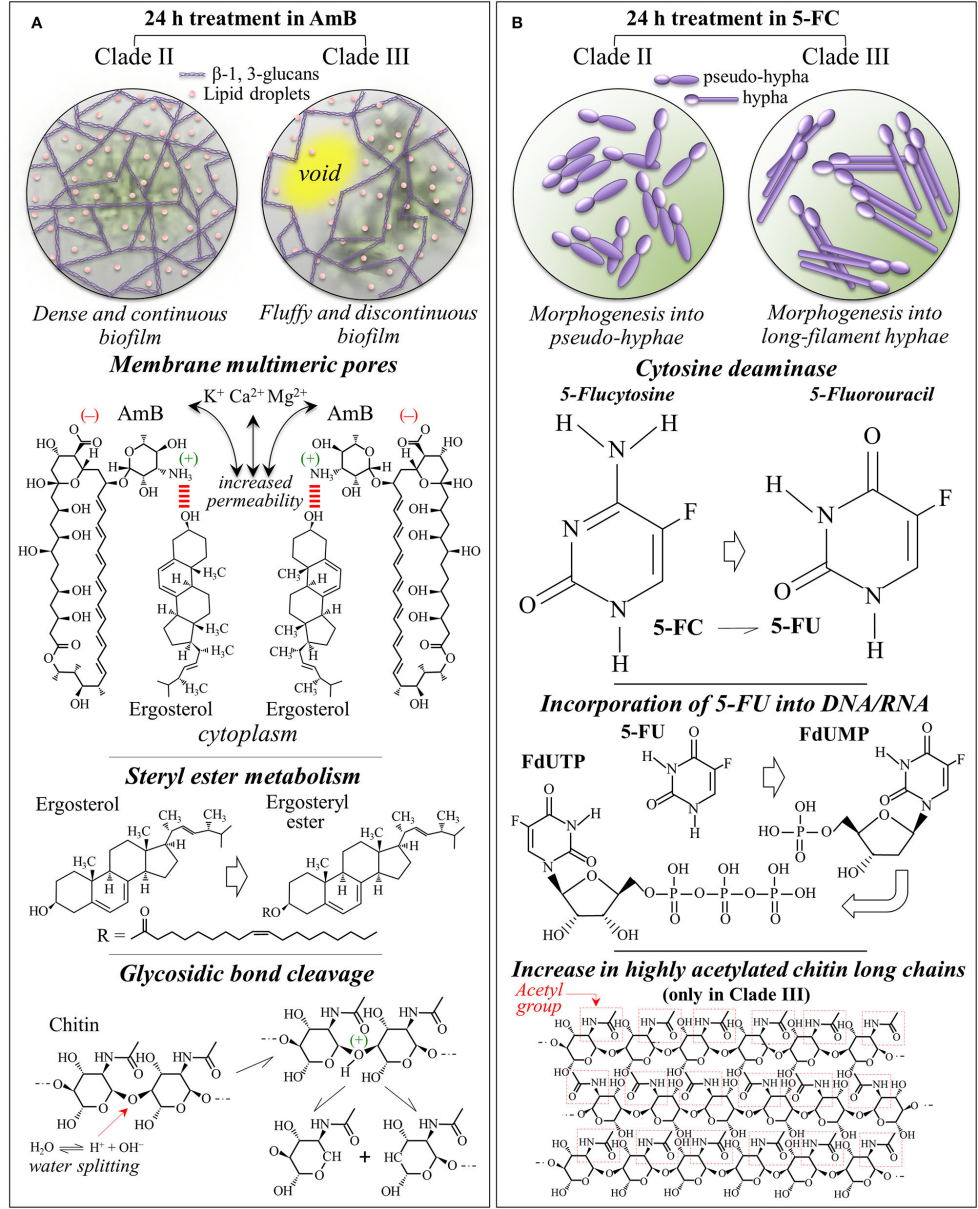
Summary of the main spectroscopic findings, including the mechanisms of action and the metabolic behaviors of Clades II and III in response to **(A)** AmB and **(B)** 5-FC 24 h treatments.

The present *in situ* Raman data gives us a chance to discuss in details the reaction of *C. auris* clades to AmB, including the actual origin and the effects of oxidative damage. Upon AmB treatment, we observed a clear split of the low-frequency ergosterol band at 594 cm^−1^ into two sub-bands at 585~588 and 603 cm^−1^, and a clear reduction of the 713 cm^−1^ band. These characteristics were consistent for both clades (cf. Figure 9A). Even assigning the entire intensity reduction of the 713 cm^−1^ band to ergosterol, this cannot necessarily represent an inhibition of ergosterol synthesis but is rather be the spectroscopic consequence of ring constraint. Structural constraints might arise from ergosterol binding with the mycosamine group of the AmB molecule and its subsequent alignment along the side of the AmB lactone ring with multiple conjugated carbon-carbon double bonds (cf. Figure 10A) (Gray et al., 2012). The invariance in the intensities of other bands related to ergosterol supports this interpretation. On the other hand, splitting of the 594 cm^−1^ ring-related band should be interpreted as a modification of the ergosterol structure and a signature of the detoxifying action developed by yeast cells to scavenge oxygen radicals (Moradas-Ferreira and Costa, 2000). The observed split indeed points at the presence of ergosteryl ester with its related fatty acid tails (cf. Figure 10A) (Krafft et al., 2005). Sterol ester metabolism in *Candida* species is known to enable yeasts to modulate the level of free sterol at different stages of growth (Mullner and Daum, 2004). The present study newly suggests that such modulation also takes place in both *C. auris* clades when yeast needs to build a biofilm in defense against AmB. In strong support of this interpretation are: (i) the observed intensification of the ester band at 2,980 cm^−1^ and of the unsaturated =CH stretching band at 3,010 cm^−1^ (cf. Figures 7A–D); and, (ii) the enhanced intensity of C=O stretching and O-C=O stretching at 1,730 and 1,750 cm^−1^, respectively, (cf. Figures 4B, 5B). Note that both these spectral features fit with the hypothesized increase in ergosteryl ester (cf. Figure 10B). *Candida* yeasts have also been reported to increase the amount of unsaturated lipids as a strategy to minimize the effect of interdigitated phases (Vanegas et al., 2012). Moreover, the observation of strong lipid signals outside the yeast cells (cf. Raman maps in Figures 8C,D) suggests an intensification in the production lipid droplets composed of triacylglycerides and steryl esters (Leber et al., 1994). Steryl ester in yeast contains a high content of fatty acids (i.e., accounting for ~75% of the total sterol content) (Madyastha and Parks, 1969; Hunter and Rose, 1972). Chang et al. (2015) have reported that trapping of endogenous toxins (i.e., self-secreted proteins and glycoproteins) by lipid droplets represents a self-resistance mechanism and acts to quench the production of reactive oxygen species. Those researchers clearly showed that, in *Candida* yeasts, neutral lipids and lipophilic agents of the droplets trap toxins and quench the formation of reactive oxygen species. Based on the above interpretation and the present results, one could argue that oxygen radicals were produced by the clades in response to the formation of toxins produced upon AmB treatment. Yeast cells then attempted to escape oxidative damage by compartmentalizing toxins into lipid droplets with the consequent sequestration of oxygen radical formation. In sum, the diffuse signals of ergosterol detected by Raman molecular imaging suggest the following events in reaction to AmB interdigitation stress: (a) stimulating the formation of lipid droplets, and (b) supporting the generation of ergosteryl ester and, possibly, of other neutral lipids. Regarding the unsolved dualism between occurrence of AmB autoxidation or cell-produced toxic radicals in response to AmB, note that the present Raman results could not support the thesis of AmB autoxidation, since no difference could be found between AmB Raman signals before and after interactions with *C. auris* clades (cf. AmB spectrum in Figure 6D with the strong and sharp AmB signals in Figures 4B, 5B). The extensive formation of lipid droplets and the Raman signatures for ergosteryl ester and elongated fatty acid tails point instead at a detoxifying action developed by yeast cells to scavenge self-produced oxygen radicals.

Also, the trend observed for the Raman signals of chitin, which represent structural variations in the chitin structure, cannot be simply explained by molecular binding between AmB and ergosterol molecules. The clear increase in chitin content found for both clades exposed to AmB is likely related to a metabolic reaction of the yeast cells to counteract the effect of the drug. However, the trend observed for the chitin spectroscopic markers clearly differed between Clades II and III. In particular, the split of the 1,112 cm^−1^ band into two sub-bands at 1,100 and 1,107 cm^−1^ in Clade III could be interpreted as a fingerprint for the formation of allomorphs with symmetric stretching vibrations from glycosidic C-O-C groups (Schenzel and Fischer, 2001). Another possible explanation for the chitin band split is glycosidic bond breakage, which can only occur in the presence of radical species (cf. Figure 10A) (Cumpstey, 2013). Note that also the clear intensity decrease of the 2,995 cm^−1^ band in Clade III (cf. Figure 7E), which is quite pronounced only in highly crystalline chitin (Ando et al., 2021), gives support to the hypothesis of crystalline chitin structure. This spectral feature, which is only peculiar to Clade III, is likely related to the formation of an extra-cellular matrix in the so-called “fluffy structured” colony, which protects the yeast community in an early stage of biofilm development (cf. Figure 10A) (Palkova and Vachova, 2006). Constructed with both polysaccharides and proteins, this extra-cellular matrix contains empty chambers, enabling the fluffy colony to quickly occupy a relatively large territory with a relatively small number of cells. This interpretation appears supported by microscopic observations on Clade III morphology after AmB exposure and related Raman maps (cf. Figures 1f, 9D). It is thus suggested that Clade III attempts a survival strategy different from Clade II under AmB environmental stress.

Additional metabolic reactions related to the structure of polysaccharides could be found in the increased glucan content and the relative enrichment in β-1, 3–glucans. Such major changes in cell-wall structure point at regulatory mechanisms aimed at compensating for drug-induced damages of cell walls and/or extracellular matrix structures. The present Raman study substantiated for the first time a difference in the population of inter and intramolecular hydrogen bonds between different clades treated with AmB. While the degree of acetylation was similarly enhanced upon AmB treatment in both clades (cf. Amide I bands), only Clade III showed a 30% decrease in intermolecular-to-intramolecular hydrogen bond ratio as compared to the as-cultured clade. These spectroscopic differences are substantiated by optical micrographs, as well as by chitin and Amide I Raman maps in Figures 8C,D (cf. labels in inset). Consistent with the ergosterol map, the chitin distribution showed the formation of a biofilm structure more continuous and denser in Clade II than in Clade III. Note that this interpretation matches the view of fluffy colony proposed above for Clade III. Biofilm formation has been reported as a strain-dependent trait in *C. auris* and strongly associated with type and phenotypic behavior of specific isolates (Singh et al., 2019). The isolates from both clades examined in this study showed a high degree of association (cf. micrographs in Figure 1). Aggregative phenotypes were indeed classified as stronger biofilm producers among colonizing isolates of *C. auris* (Singh et al., 2019). Aggregation and biofilm formation appeared stronger (or faster) in Clade II, which thus possesses a higher degree of ecological fitness and survivorship. As usual in aggregative-colonizing isolates, however, the examined isolates from both clades commonly showed a comparatively strong susceptibility to AmB (Singh et al., 2019).

### Raman Insights Into Yeast Responses to 5-FC Exposure

Although the exact mechanism of action of 5-FC is yet the object of investigation (Gsaller et al., 2018), it is believed that 5-FC induces in yeast cells a direct mechanism of competitive inhibition of purine and pyrimidine uptake and an indirect mechanism of intracellular metabolism to 5-FU (Polak and Scholer, 1975). After penetrating the cells *via* cytosine permease, 5-FC is promptly metabolized to 5-FU. 5-FU is then converted into 5-fluorouridine monophosphate (FUMP) either through direct or indirect phosphorylase reactions. In successive steps, FUMP undergoes further phosphorylation into fluorouridine diphosphate (FUTP) and further converted into either active metabolite fluorodeoxyuridine triphosphate (FUTP) upon ribonucleotide reductase (Longey et al., 2003). These active metabolites become misincorporated into both RNA and replicating DNA as a consequence of their strong similarity with uridine-5'-triphosphate/2'-deoxythymidine-5'-triphosphate. This process, which is schematically shown in Figure 10B, leads to genome instability and inhibits the synthesis of both DNA and RNA leading to unbalanced cell growth and death. Machon et al. (2021) described a method based on liquid chromatography coupled with high-resolution mass spectrometry suitable for the simultaneous determination of the ten anabolic metabolites (nucleoside, nucleotide and sugar nucleoside) of 5-FU. This composite analytical method successfully measured the proportion of different anabolic metabolites of 5-FU in cellular structures, thus confirming their incorporation in DNA and RNA in agreement with the draft in Figure 10B.

The present Raman data taken at 24 h exposure basically confirmed this action scenario with revealing the main 5-FU (and not 5-FC) bands inside the yeast cells (cf. average spectra in Figures 4C, 5C, and 5-FU Raman images in Figures 9E,F) and the almost complete disappearance of Raman markers of deoxycytidine and deoxythymidine ring bands (cf. maps in Figures 9E,F). Unlike the case of AmB treatment, 5-FC treatment did not induce biofilm formation, but an extensive morphological transition into pseudohyphae and hyphae could be observed in Clades II and III, respectively (cf. Figures 1c,d). Filamentous growth transition in *C. auris* has been observed upon growth in NaCl-rich yeast extracted-peptone-dextrose medium (Wang et al., 2018), upon deletion of heat shock molecular chaperone protein (Kim et al., 2019), or upon passaging through a mammalian host (Yue et al., 2018). The present study showed that treatment with 5-FC induced hyphal-like phenotype transition in both Clades II and III, which from the one hand helps explain their success as highly virulent pathogens and, on the other hand, gives hints on their emergent resistance to this drug.

In addition to confirming the 5-FC mechanism of action, Raman spectroscopy revealed important details as well as differences in the metabolic reactions of different clades to the drug. While both clades reacted to 5-FC exposure with a similar overproduction of ergosterol (likely in an attempt to reduce membrane permeability) and chitin (with a clear increase in the intermolecular-to-intramolecular hydrogen bond ratio), only Clade III produced its enhanced fraction of chitin structure with a high degree of acetylation (cf. Figure 10B). This effect represents a remarkable “chemical” trick for reducing permeability while further increasing membrane stiffness beyond the mere effect of a fractional increase (Motta de Moura et al., 2011). Another remarkable “chemical” ability of Clade III over Clade II was represented by its capacity to increase by nearly a factor 2 the chain length of the produced fatty acids (i.e., the intensity ratio of C-H(CH_2_) to C-H(CH_3_) stretching bands, I_2850_/I_2935_) in response to 5-FC treatment. Longer fatty acids are indeed more rigid and can greatly reduce membrane fluidity and permeability (Cavalheiro and Teixeira, 2018; Tyler et al., 2019). Because of its “chemical skills”, Clade III could be extremely efficient in developing resistance to 5-FC treatments.

### Implications of Hyphal Morphogenesis Upon 5-FC Treatment

Wilson et al. (2016) documented the role of hyphal morphogenesis on host cell damage in *C. albicans* and showed that both yeast and hyphal cells are required for reaching high virulence. In systemic infections, neither yeast-locked strains nor hyperfilamentous mutants are fully virulent, but it is their combination and morphogenic switching capacity that leads to the highest virulence with the two forms fulfilling specific functions during infection: dissemination *via* blood stream by yeast, adhesion and invasion of host cells by filamentous hyphae. Several independent studies have proposed a direct correlation between the formation of *C. albicans* hyphae and an enhanced capacity for tissue invasion, damage, and virulence, while the capacity of pseudohyphal forms is yet unclear (Lo et al., 1997; Saville et al., 2003; Gow et al., 2011). In a study on peripheral blood mononuclear cells (Mukaremera et al., 2017), yeast cells were found to induce the generation of more inflammatory cytokines than hyphae, while pseudohyphae induced intermediate cytokine levels. This indicates that immune recognition targets more severely yeast cells, while filamentous hyphal cells induce an altered immune response (Gantner et al., 2005; Van Der Graaf et al., 2005; Gow et al., 2011; Moyes et al., 2011). *C. auris* has also been reported to exhibit cellular polymorphism to produce pseudohyphal cell growth in the presence of genotoxic stress induced by hydroxyurea (Bravo et al., 2020); growth in high salt concentrations also induced cell elongation (Kim et al., 2019). Filamentous or elongated morphologies were also observed in *C. auris* populations retrieved from murine infection (Yue et al., 2018; Fan et al., 2021). However, hyphal morphogenesis did not occur in a number of circumstances that induce hyphal growth in *C. albicans* (Wang et al., 2018). The effect of drugs causing genotoxic stress, such as hydroxyurea or methyl methanesulfonate, can trigger an arrest of genetic replication *via* a cell cycle checkpoint (Pardo et al., 2017). Our present data are in agreement with those reported in Bravo et al. (2020), showing that 5-FC (converted into fluorouracil in the *Candida* yeast cells) perturbed RNA and DNA biosynthesis causing the growth of pseudohyphal cells in Clade II. By showing morphological differences between Clade II and Clade III treated under the same conditions, we also confirmed that *C. auris* filamentation is strain dependent (Bravo et al., 2020). However, a new result here is that LSEM 3673 (South African) Clade III was converted into a fully developed hyphal morphology upon 5-FC treatment. This result confirms the capacity of hyphal switching for *C. auris* so far only observed for yeast passaging through a mammalian body (Yue et al., 2018) or in genetically manipulated morphogenic mutants (Santana and O'Meara, 2021). This latter study revealed a dysregulated chitinase expression, attenuated virulence, and altered antifungal susceptibility in morphogenic mutants. Parallel to genomic analyses, the present study adds molecular structure insight into an enhanced fraction of chitin with high intermolecular-to-intramolecular hydrogen bond ratio and a high degree of acetylation (cf. Figure 10B) for LSEM 3673 Clade III.

## Conclusion

A twofold spectroscopic approach was followed in performing Raman analyses of *C. auris* Clades II and III before and after exposure to AmB or 5-FC. First, tens of spectra were collected with a 20x optical lens to cover relatively large areas of the clade cultures (in the order of mm^2^) with high spectral resolution, and their average computed for each clade and drug treatment. The obtained average spectra were deconvoluted according to a specifically tailored machine-learning algorithm linked to a database of Raman spectra from elementary molecules. Then, millions of spatially resolved spectra were taken through mapping on more limited areas of the cultures (in the order of 10^−2^ mm^2^) and converted into Raman hyperspectral images at wavelengths representing selected lipid, polysaccharide, and DNA molecules. These two complementary procedures gave consistent results and unfolded important differences in the way different clades react to different antifungal drug treatments with important implications on cellular morphogenesis, virulence, and drug resistance.

Besides confirming the main mechanisms of action of the two drugs on the studied clades, as reported in the literature, Raman analyses/imaging revealed major changes in cell-wall structures, which were interpreted as the results of regulatory mechanisms to compensate for structural alterations and damages inflicted by drug treatments. Both clades responded to AmB treatment by building a biofilm; however, the biofilm structures were different for different clades: Clade II formed a dense and continuous biofilm structure, while Clade III built an extra-cellular matrix with a “fluffy” and discontinuous structure. On the other hand, no biofilm formed in any clade upon treatment with 5-FC, but fungal morphogenesis with pseudo-hyphal and hyphal transitions being observed in Clade II and Clade III, respectively. Clade III showed a superior capacity to chemically tailor chitin structure with a high degree of acetylation and fatty acids networks with significantly elongated chains, both structural characteristics greatly reducing membrane permeability to the drug. Future Raman research on susceptibility and stress responses of *C. auris* clades to antifungal drugs may help to identify new cellular targets for treating invasive fungal infections and to develop novel clinical solutions to counteract the spread of these alarming pathogens.

## Data Availability Statement

The raw data supporting the conclusions of this article will be made available by the authors, without undue reservation.

## Author Contributions

GP, MK, OM, TY, NK, IN, and KM contributed to conception and design of the study. TNakay, HI, TAs, NM, TAd, and EO performed the experiments. GP and TNakat organized the database. WZ performed the statistical analysis. WZ and EM supervised. GP wrote the first draft of the manuscript. All authors contributed to manuscript revision, read, and approved the submitted version.

## Funding

This work was supported by the Strategic Foundational Technology Improvement Support Operation 2019 of the Japanese Government, a Grant-in-Aid from Tokuyama Science Foundation 2020 (T.A.), the JSPS KAKENHI Grant Number 22K10128, the JST SPRING Grant Number JPMJSP2107 (H. I., 2022), and the Young Scientists at Kyoto Prefectural Public University Corporation (T. A., 2021).

## Conflict of Interest

The authors declare that the research was conducted in the absence of any commercial or financial relationships that could be construed as a potential conflict of interest.

## Publisher's Note

All claims expressed in this article are solely those of the authors and do not necessarily represent those of their affiliated organizations, or those of the publisher, the editors and the reviewers. Any product that may be evaluated in this article, or claim that may be made by its manufacturer, is not guaranteed or endorsed by the publisher.

## Footnotes

^1^https://www.cdc.gov/drugresistance/pdf/threats-report/candida-auris-508.pdf; (accessed January 1, 2022).
